# New pharmacotherapies for the erythropoietic protoporphyrias: an analysis of trial protocols from a patient perspective

**DOI:** 10.1186/s13023-025-04170-9

**Published:** 2025-12-29

**Authors:** Cornelia Dechant, Sebastian Wäscher, Francesca Granata, Nicole Gusset, Mårten Pettersson, Mehmet Hakan Aksözen, Marc Höglinger, Rocco Falchetto, Jasmin Barman-Aksözen

**Affiliations:** 1 International Porphyria Patient Network, Hegarstrasse 3, Zurich, 8032 Switzerland; 2 Institute for Social Ethics, Centre for Ethics of the University of Zurich, Zollikerstr. 117, Zurich, 8008 Switzerland; 3 University Research Priority Program “ITINERARE – Innovative Therapies in Rare Disease”, University of Zurich, Zurich, Switzerland; 4 Fondazione IRCCS Ca’ Granda Ospedale Maggiore Policlinico, S.C Medicina ad Indirizzo Metabolico, 20122 Milano, Italy; 5 SMA Schweiz, Alpenstrasse 76, Heimberg, 3627 Switzerland; 6 Winterthur Institute of Health Economics, School of Management and Law, Zurich University of Applied Sciences, Winterthur, 8401 Switzerland; 7 Institute of Laboratory Medicine, Swiss Reference Centre for Porphyrias, Municipal Hospital Zürich Triemli, Zurich, 8063 Switzerland; 8 Division of Metabolism and Children’s Research Center, University Children’s Hospital, Zurich, CH-8032 Switzerland

**Keywords:** Erythropoietic protoporphyria, Rare diseases, Afamelanotide, Dersimelagon, Bitopertin, Cimetidine, Trial design, Placebo, Ethical issues, Patient perspective

## Abstract

**Background:**

The erythropoietic protoporphyrias (EPP) are a group of ultra-rare (1:100.000) inborn errors of the heme biosynthesis characterised by painful phototoxic reactions in tissue exposed to visible light. Afamelanotide is the only approved treatment for EPP and effectively prevents phototoxic reactions and improves the quality of life of the patients. In the past years, several new potential treatment options for EPP have been identified, some of which are currently under investigation in clinical trials. While these developments could improve patient care, it is important to know how safety and efficacy of drug candidates compare to the existing treatment, i.e. afamelanotide.

**Methods:**

We identified pharmacotherapies (leaving out, for example, topical applications such as sunscreens or supplements such as iron) which are currently (that is, within the last 5 years) evaluated for EPP from clinical trial registries and investigated whether the trial designs allow a comparison of their treatment effects with each other and with afamelanotide. Therefore, we analysed the clinical trial protocols with emphasis on their trial designs, efficacy outcome measures, inclusion and exclusion criteria, safety aspects and, if available, published results.

**Results:**

Our search in the clinical trials registries retrieved 29 trials that included patients with EPP. From these, we identified 16 clinical trials evaluating afamelanotide and three new pharmacotherapies, i.e., dersimelagon, bitopertin and cimetidine. Safety and efficacy of all new pharmacotherapies are currently being investigated against placebo-control groups or against baseline. Because of differences in the outcome measures and included patient populations, the results of the trials cannot be directly compared. Moreover, methodically challenging aspects and ethical issues were identified in some of the trial protocols.

**Conclusion:**

Efficacy and safety of currently investigated treatments for EPP are not directly comparable. We wrote this manuscript as a call to action to Principal Investigators, Ethical Review Boards, Regulatory Authorities and the sponsors of trials because we are convinced that trials directly assessing new pharmacotherapies against afamelanotide would be the more informative, and methodological and ethically sounder trial design.

**Clinical trial number:**

Not applicable.

**Supplementary Information:**

The online version contains supplementary material available at 10.1186/s13023-025-04170-9.

## Introduction

**Fig. 1:**
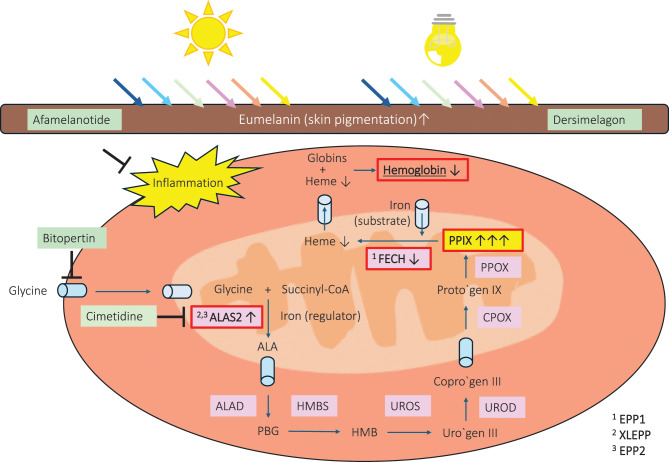
Subtypes of erythropoietic protoporphyria (EPP) and mode of action of currently investigated pharmacotherapies: heme is built in all cells in eight enzymatic steps from the substrates glycine, succinyl-CoA and iron. The three subtypes of EPP affect the heme biosynthesis in the erythrocytes and lead to the accumulation of protoporphyrin IX (PPIX), the last precursor of heme: EPP1 (OMIM #177000) is caused by a partial deficiency of ferrochelatase (FECH), the last enzyme of the heme biosynthetic pathway; XLEPP (OMIM #300752) is caused by gain-of-function mutations in the first and rate limiting enzyme of the erythroid heme biosynthesis, delta-aminolevulinate synthase 2 (ALAS2). Mutations in CPLX, which regulates the activity of ALAS2, cause the rare subtype EPP2 (OMIM #618015). PPIX absorbs the energy of the visible light range and causes painful phototoxic reactions and burn injuries in tissue exposed to visible light. Around 5% of the patients develop a cholestatic liver failure, caused by the hepatotoxic properties of PPIX which is excreted via the liver. In addition, most patients show disturbances in their iron metabolism and/or a microcytic, hypochromic anemia that mimics iron deficiency. Until now, the only approved therapy for EPP is afamelanotide, a small peptide analogon of the alpha-melanocyte stimulating hormone that binds to the melanocortin 1 receptor (MC1R) on melanocytes. It acts by increasing eumelanin (brown skin pigment) synthesis, which prevents visible light from reaching the blood vessels, and, in addition, has strong anti-inflammatory properties, which is assumed to contribute to the therapeutic effect in EPP. Currently investigated pharmacotherapies are dersimelagon, bitopertin and cimetidine. Dersimelagon is a small molecule which, like afamelanotide, binds to the MC1R. Bitopertin is an inhibitor of the glycine transporter 1 (GlyT1) and decreases the uptake of the heme substrate glycine into the cell. Investigating cimetidine is based on the hypothesis that it inhibits the activity of ALAS2

The erythropoietic protoporphyrias (EPP) are a group of ultra-rare (1:100.000) inborn errors of metabolism in which protoporphyrin IX (PPIX), a precursor of the red blood dye heme, accumulates in the red blood cells and blood vessels (Fig. 1) [1–5]. Most patients with EPP show a moderate anemia and/or disturbances in their iron metabolism [6]. In addition, around 5% of the patients develop a life-threatening cholestatic liver failure, caused by the hepatotoxic properties of PPIX which is excreted via the liver [7–9]. However, the most burdensome symptom in all three subtypes of EPP is a debilitating intolerance to sunlight and certain artificial light sources: PPIX absorbs the energy of the visible light range and within minutes causes phototoxic reactions and burn injuries in the exposed tissue [10–13]. The associated severe pain is exacerbated by environmental factors such as heat, wind and artificial light, can last for days and does not respond to analgesics, including opioids [13–19]. From early childhood, patients with EPP have an anxiety to be exposed to light and avoid situations in which they might be at risk of developing symptoms, which has a negative impact on their overall social, educational and occupational development, as well as their mental health [11, 20–25].

Afamelanotide is the first treatment for EPP for which safety and efficacy in preventing phototoxic reactions and increasing the time spent in sunlight has been demonstrated in randomised, placebo-controlled clinical trials (RCTs) [26]. In 2014, the European Medicines Agency (EMA) recommended approval of afamelanotide for treating adult patients with EPP in the EU, followed by approval for adults in the USA (2019) and in Australia (2020). Since then, safety, effectiveness and high treatment satisfaction with afamelanotide in EPP have been confirmed in several long-term real-world evidence studies [27–32]. Moreover, in 2021, the International Rare Diseases Research Consortium (IRDiRC) assessed afamelanotide as being “efficacious, safe and having a significant impact on the quality and/or duration of life” and included it in their list of 204 essential medicinal products for rare diseases [33]. Therefore, EPP belongs to the minority of ultra-rare diseases for which an approved and effective treatment option exists. However, considerable medical needs, such as treatment options for children and adolescents, remain unmet: because of its mode of administration as a 16 mg fixed dose slow-release subcutaneous implant, dose-adjustments of the currently approved formulation of afamelanotide are not possible, which makes it unsuitable for the treatment of children. Afamelanotide is also not sufficiently effective or suitable for all adult patients, and, being a symptomatic treatment, does not address the increased risk for EPP-related liver failure. Moreover, because of lack of reimbursement, afamelanotide is not yet available to patients in many countries, despite being the only approved treatment for EPP [34]. Therefore, new treatment options for EPP which address the above-mentioned issues are desirable, especially treatments for children and adolescents which are the most vulnerable and critically affected age groups [11, 20, 24]. In the past years, new insights into the underlying disease pathology and the development of disease models, such as genetically modified EPP cell lines, made the discovery of new drug candidates and screening for repurposed drugs more feasible, some of which are currently being investigated in clinical trials [35].

While new treatments might lead to improved patient care, it is important to know how the safety and efficacy of drug candidates compares to the existing treatment, i.e., afamelanotide. However, until now, a detailed analysis on how well afamelanotide and currently investigated drug candidates for EPP can be compared is lacking. Therefore, for our study, we screened clinical trial registries for treatments that are currently (defined as withing the last 5 years) under investigation for EPP and analysed the trial protocols of the identified pharmacotherapies (leaving out, for example, topical applications such as sunscreens and dietary supplements such as iron). For our analysis, we focused on the trial designs, efficacy outcome measures, inclusion and exclusion criteria and, if available, published results. In addition, we discuss safety considerations and methodological and ethical issues identified during the analysis and outline potential solutions from a patient perspective.

## Material and methods

The presented study is an analysis of publicly available documents from clinical trial registries and, where available, peer-reviewed publications and reports from regulatory agencies (which assess the safety and efficacy of therapies prior to their approval) and Health Technology Assessment (HTA) bodies (which after regulatory approval evaluate the costs and benefits of a treatment to inform national reimbursement decisions). The aim of the study was to assess how well the results of trials investigating afamelanotide and new pharmacotherapies for EPP can be compared. Therefore, the trial protocols were systematically analysed using the PICO scheme, i.e., by assessing the included population, intervention, comparators and outcomes of each trial [36]. In addition, insights from the patients involved in the analysis are provided to supplement aspects of daily living with EPP.

### Researcher characteristics

The researchers involved in the study are either members of the International Porphyria Patient Network (IPPN) and/or academic researchers. The IPPN was founded in 2016 and is a not-for-profit organisation of patients and carers with professional backgrounds in medicine, biochemistry, molecular biology, economics and other relevant expertise. Its members were involved in different stages of the development and regulatory approval and/or subsequent HTA procedures of afamelanotide and other pharmacotherapies for EPP and related diseases, collectively called the porphyrias [37–40]. All members of the IPPN who are patients with EPP are currently treated with afamelanotide. Two members of the IPPN and authors of this article (FG and JBA) are biomedical researchers and part of academic collaborations that investigate new drug targets and treatment options for EPP, one of which, bitopertin, is currently under investigation in clinical trials [41–46]. Two academic researchers (SW and MH) not involved in the IPPN have substantially reviewed the manuscript, critically reviewed the arguments and methodological rigorousness. In addition, NG, a researcher and patient advocate for other rare diseases, was consulted to obtain a patient perspective from outside of the porphyrias.

### Identification of clinical trials in EPP

**Fig. 2:**
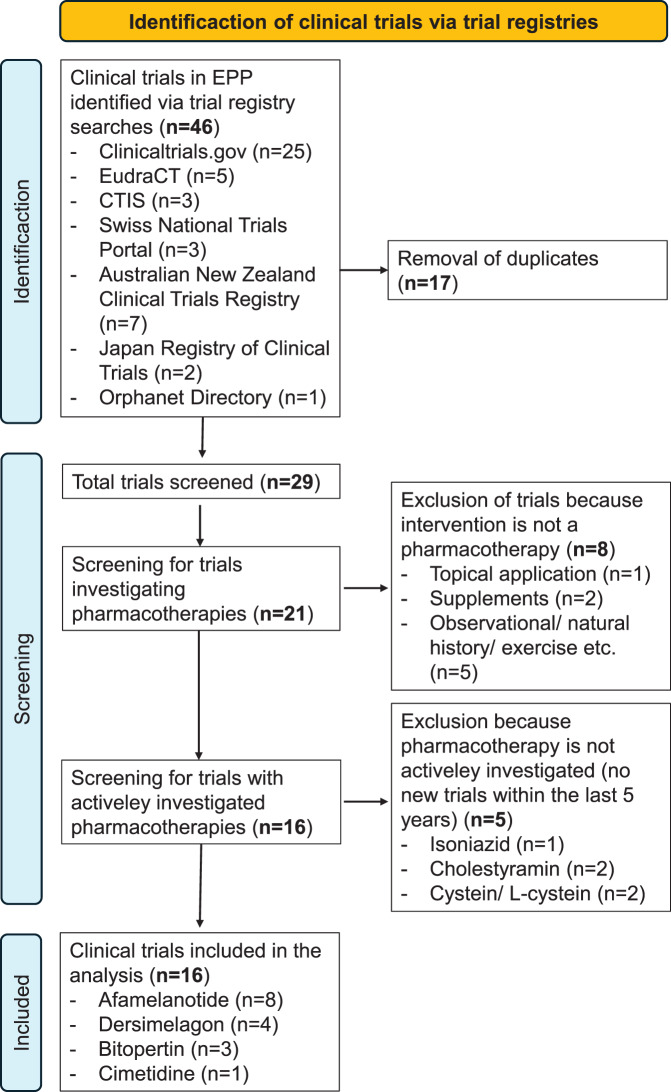
PRISMA flow chart for the systematic search for clinical trials in EPP

In a first step, CD and JBA screened the publicly accessible online registries listed in table S1 for clinical trials and studies that include subjects with EPP. The systematic search for clinical trials in EPP and screening of the results is depicted in Fig. 2. The search was conducted between August to November 2024 and repeated in February 2025, the search terms used were “erythropoietic protoporphyria” and “protoporphyria” for condition/disease. We included trials with patients with EPP from all phases of drug development, i.e., proof-of-concept and dose-finding studies (phase II), studies assessing efficacy and safety (phase III) and studies monitoring the long-term safety and effectiveness of drugs under real-world conditions (phase IV) (Table S2).

### Focus on currently investigated pharmacotherapies

We were interested in pharmacotherapies for treating EPP which are actively under investigation. Therefore, we (1) excluded clinical trials that were not followed by further trials for ≥5 years after conclusion and terminated clinical trials. (2) We further excluded trials investigating topical applications such as sunscreens or supplements such as beta-carotene, because of lack of robust evidence for their effectiveness [47, 48]. (3) We also did not include trials investigating iron for treating EPP. Iron is both a substrate and regulator of the erythroid heme biosynthesis, and iron supplementation has contrasting effects on the different subtypes of EPP: While in XLEPP, iron supplementation leads to a decrease in PPIX, studies in EPP1 demonstrate that iron supplementation increases PPIX and phototoxicity [6, 46]. For the analysis, one researcher (JBA) prepared one table for each pharmacotherapy listing, after removal of duplicates, all identified clinical trial protocols in chronological order (most current first) (Table S3).

### Analysis

Two researchers (CD and JBA) independently analysed the trial protocols of currently investigated pharmacotherapies according to the categories of the PICO framework, i.e., the population (e.g., subtypes, age ranges, eligible sexes, inclusion and exclusion criteria), intervention (e.g., dosing, trial durations, mode of administration), comparator (e.g., placebo, active control), outcome measures (e.g., primary and secondary outcome measures, others) and the study design [36]. For the assessment of the existing pharmacotherapy afamelanotide, the analysis focused on the pivotal study, as its efficacy endpoints and overall trial design have been accepted for regulatory approval [26]. For the analysis of the new pharmacotherapies, we focused on the most advanced clinical trials. The information of these trials was supplemented with aspects from other trials if providing additional information such as having a broader age range, different inclusion or exclusion criteria etc. We also compared the instruments used in the trials to assess quality of life (QoL) and patient reported outcomes. We did not systematically compare safety endpoints: We assessed that the evaluation of the safety of a new pharmacotherapy is mostly based on the retrospective analysis of adverse events that occurred during and/or after the trial, and we considered that there is not yet sufficient long-term data available to reasonably compare the safety outcomes of the currently investigated pharmacotherapies [49, 50]. Instead, we discussed safety-related considerations when comparing the new pharmacotherapies versus afamelanotide. For the analysis, aspects assessed as potentially having an influence on the comparability of the trial outcomes were marked (Table S3) and discussed amongst the members of the IPPN. For the interpretation of some of the identified issues and methodical considerations, the independent academic researchers were consulted. Some aspects were further explored by additional literature searches. The results of the analyses were compared, and differences were discussed until an agreement was reached.

## Results

### Currently investigated pharmacotherapies for EPP

**Fig. 3:**
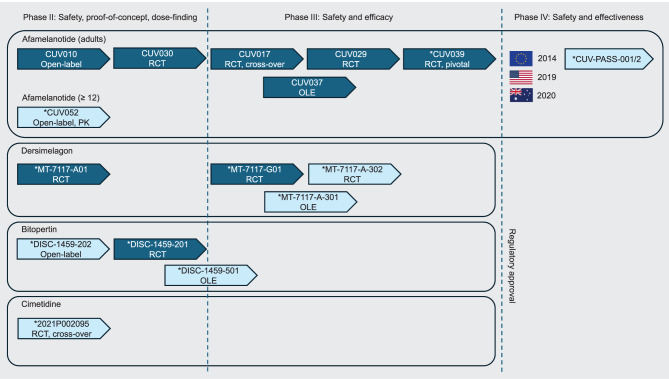
Schematic representation of the identified clinical trials of pharmacotherapies currently investigated for treating EPP. All 16 trials were identified from the trial registries listed in Table S1. Concluded trials are depicted by dark blue arrows and currently ongoing trials by light blue arrows (February 2025). For each trial, the study ID as provided by the sponsor of the trial and information on the design is provided. Phase II studies focus on aspects such as safety, dose-finding and a general proof-of-concept, phase III studies aim to demonstrate safety and efficacy for regulatory approval (marketing authorisation) and phase IV studies prospectively collect information on aspects such as the safety and effectiveness of treatments under real-world conditions. Actual time periods and trial durations are not represented proportionally. Trials and studies marked with (*) are included in the further analysis; RCT: randomised controlled trial; PASS: post-authorisation safety and effectiveness study; OLE: open-label extension study; PK: pharmacokinetics. Country flags: wikimedia commons licence

Our search in the publicly accessible trial registries, after removal of duplicates, retrieved 29 trials, including open-label (OL), open-label extension (OLE) and post-authorisation safety and effectiveness studies (PASS) (see Fig. 2). From these, we identified four pharmacotherapies for treating EPP that are currently investigated, in collectively 16 clinical trials (concluded and ongoing), i.e., dersimelagon ( = MT-7117), bitopertin and cimetidine, and the pharmacokinetics of afamelanotide in patients from age 12 (Fig. 3).

### Characteristics of the investigated pharmacotherapies

While afamelanotide and dersimelagon are symptomatic treatments, bitopertin and cimetidine aim to decrease blood PPIX concentration which makes them potentially causative treatment options (Fig. 1) [45, 51–55]. Unlike the currently approved formulation of afamelanotide which needs to be administered subcutaneously every two months, dersimelagon, bitopertin, and cimetidine are orally available which allows dose-adjustments and therefore make clinical trials in children more feasible. Bitopertin and cimetidine are repurposed drugs that have been previously investigated (bitopertin) and/or approved (cimetidine) for treating other disorders [45, 56].

### Comparison of the trial protocols of the investigated pharmacotherapies

We compared the trial protocols of the four currently investigated pharmacotherapies for treating EPP regarding their overall trial design and according to the PICO scheme, i.e., included study populations, interventions, comparators and efficacy outcome measures. [36] The main findings are summarised in Table 1, and the detailed results as extracted from the trial protocols can be found in the Table S4.

**Table 1:** Comparison of the trial protocols of the four currently investigated pharmacotherapies for treating EPP, summary of the main findings (for full details, see Table S4)

	Afamelanotide	Dersimelagon	Bitopertin	Cimetidine
**Population (inclusion and exclusion criteria)**				
Subtypes (EPP1, EPP2, XLEPP)	All	All	All	All
Age range	Pivotal study: ≥18 Pharmacokinetics: ≥12 to 70	≥12 to 75	≥18	≥15
Sex	Male and female	Male and female	Male and female	Male and female
Requirement to cease treatment with afamelanotide	n.a.	Requirement to cease treatment with afamelanotide	Requirement to cease treatment with afamelanotide	
Requirement to expose to sunlight during the clinical trial		Requirement to expose to sunlight during the clinical trial		Requirement to expose to sunlight during the clinical trial
Liver health	Exclusion criterion: EPP patients with significant hepatic involvement	Exclusion criterion: EPP patients with significant hepatic involvement	Exclusion criterion: EPP patients with significant hepatic involvement	Exclusion criterion: EPP patients with significant hepatic involvement
Skin cancers	Exclusion criteria: Skin cancer or premalignant skin lesions.	Exclusion criteria: Skin cancer or premalignant skin lesions.		
Anemia			Exclusion criterion: Hemoglobin < 10 g/dL at Screening	
Depression and suicidal ideations			Exclusion criterion: Depression or suicidal ideations (DISC-1459–501, OLE)	
Previous exposure to study drug		Exclusion criterion: Subjects who participated in any previous dersimelagon clinical studies		Exclusion criterion: Use of cimetidine within the past 3 months at screening
**Intervention**				
Substance class	Peptide	Small molecule	Small molecule	Small molecule
Effect	Symptomatic	Symptomatic	Causative	Causative
Mode of action	Binding to the melanocortin 1 receptor (MC1R)	Binding to MC1R	Inhibitor of glycine transporter 1	Inhibitor of ALAS2
Administration	Approved formulation: 16 mg slow-release subcutaneous implant formulation, every 60 days	Oral, once daily	Oral, once a day	Oral, twice daily
**Comparator**				
Comparator main trial	Placebo	Placebo	Placebo	Placebo, crossover study
**Outcomes**				
Primary outcome measure(s)	Time in sunlight without pain	Time outdoors until prodromal* symptoms develop	Percent change from baseline in whole blood protoporphyrin levels	Percent change from baseline in erythrocyte total protoporphyrin levels
Secondary outcome measure(s), selection	Combined Sun Exposure and Phototoxic Pain. Maximum severity of phototoxic reactions Number of phototoxic reactions	Number and severity of pain events	Time in sunlight without pain Time until prodromal* symptoms develop Maximum severity of phototoxic reactions	Time until prodromal* symptoms develop Maximum severity of phototoxic reactions
QoL and PRO measures	DLQI EPP-QoL.	PGIC PROMIS-57	PGIC	PROMIS-57

*Prodromal symptoms are early warning signals for a phototoxic reaction, and include symptoms such as itching, tingling, burning and stinging; QoL: Quality of Life; PROM: Patient Reported Outcome; DLQI: Dermatology Life Quality Index; EPP-QoL: EPP Quality of Live; PGIC: Patient Global Impression of Change; PROMIS-57: Patient-Reported Outcomes Measurement Information System

### Completeness and consistency of the information from the trial registries

During the analysis, we noted inconsistencies between some of the trial protocols as deposited in the trial registries and published results and information. For example, according to the trial protocol of the phase II trial investigating bitopertin in Australia (DISC-1459–202), patients had to be at least 18 years old to be eligible for participation (Australian New Zealand Clinical Trials Registry, last updated 25 Jan. 2025, last accessed 5 May 2025). However, a conference abstract on its outcomes reports that participants from age 12 have been included in the trial [54]. Another example is the afamelanotide pharmacokinetics study in patients from age 12, which according to the information on clinicaltrials.gov is conducted in two treatment centres, one in Belgium and one in The Netherlands. However, in a company announcement which reports preliminary results of the study, three treatment centres are mentioned (location not provided) [57].

## Discussion

Our analysis of trial protocols of the four currently investigated pharmacotherapies for EPP, i.e., dersimelagon, bitopertin, cimetidine and the approved treatment afamelanotide revealed considerable differences in the included trial populations, outcome measures and mode and frequency of administration (Table 1), which limit the comparability of the trial results [58]. In addition, during the analysis, we identified ethically challenging aspects and methodological issues, some of which could be addressed by alternative trial designs as outlined under below.

### Representativeness of the trial populations

Some of the inclusion and exclusion criteria for the analysed trials likely have an impact on the representativeness of the included trial populations, i.e., the effects of the treatments are not assessed in the full range of patients in the EPP population [59–61].

#### Included subtypes of EPP

EPP is caused by pathogenic variants in three different genes associated with the heme biosynthesis pathway. (Fig. 1) Differences in the subtypes of EPP included in the clinical trials may have an influence on approval, reimbursement and treatment decisions. All subtypes are biochemically characterised by an increase in blood PPIX concentration and phototoxicity but differ in other aspects, for example, their reaction to iron supplementation (see Section “Patient perspective: Selection of a less severely affected trial population”). In the analysed trials, patient inclusion is based on a medical history of EPP and/or a biochemical diagnosis, i.e., an increase in PPIX, with or without a genetic confirmation. Therefore, no subtype of EPP was excluded from any of the trials of the four currently investigated pharmacotherapies. However, as some therapies might affect the subtypes differently, subtype-specific results should be reported in all future trials (see Section “Patient perspective: Selection of a less severely affected trial population”).

#### Age ranges and sex of the patients included in the studies

The observed differences in the age ranges of the included trial participants are likely explained by the current phase of the drug development, with earlier studies more likely to include adults only. (Table 1) For dersimelagon, bitopertin and cimetidine which are oral treatments, adjustments of the dose to lower body weights are possible and might lead to treatments for children. For afamelanotide, clinical trials in patients below age 12 will most likely not be possible with the currently approved fixed dose implant formulation. All trials include male and female patients.

#### Selection for a less severely affected trial population

**Fig. 4:**
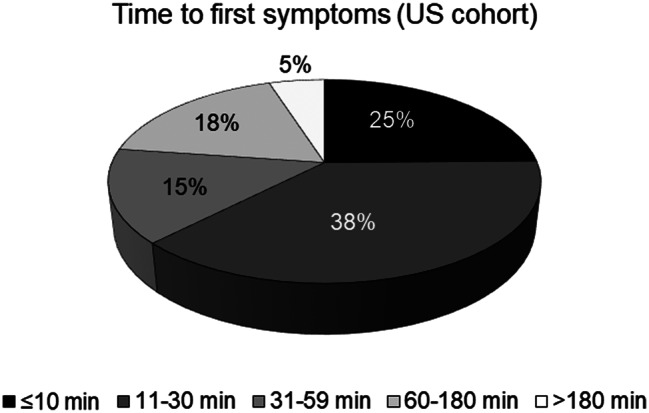
Distribution of patients according to their time in sunlight to first symptoms, US cohort (data adopted from [18])

Some of the inclusion and exclusion criteria will likely select for a less severely affected trial population as compared to the trials investigating afamelanotide: For the dersimelagon phase II and phase III clinical trials, patients are not accepted as participants if they are “unwilling or unable to go outside during daylight hours”. Moreover, according to the published trial protocol for the dersimelagon phase II trial, the participants were asked to expose themselves daily to sunlight until symptoms develop [52]. In contrast, the trials investigating afamelanotide measured the time the participants voluntarily spent in sunlight [26]. Similarly, for the cimetidine trial, patients are excluded if they are “not willing to expose themselves to light to the point of prodromal symptoms at least weekly.” Prodromal symptoms are a newly introduced concept of early warning signals for a phototoxic reaction, and include symptoms such as itching, tingling, burning and stinging. While prodromi might act as a proxy for a full reaction, the concept is not yet sufficiently established (see Section “Patient perspective: Selection of a less severely affected trial population”). In addition, the trials investigating dersimelagon and bitopertin are placebo-controlled and do not allow concurrent treatment with afamelanotide. Besides being ethically questionable (see Section “Patient perspective: Selection of a less severely affected trial population”), these requirements might prevent more severely affected patients from participating in the trials: Most patients with EPP must function in daily life, for example go to work or bring their children to kindergarten, and more severely affected individuals will likely not risk becoming incapacitated because they are taking part in a clinical trial that expects from its participants to expose themselves to sunlight until they develop symptoms. Indeed, a previous study by Balwani et al. [18]. characterising the entire US cohort found that most patients with EPP have between 11 to 30 minutes time in sunlight before they develop symptoms. (Fig. 4) In comparison, in the phase II trial investigating dersimelagon (which was conducted in the US), the baseline time to first symptom was around 60 minutes [52]. This indicates that, on average, the trial participants were less severely affected than the overall US patient cohort.

##### Patient perspective: selection of a less severely affected trial population

From a patient perspective, it could be justified to select less severely affected individuals for clinical trial programmes in EPP as they might be more daring to test their tolerance to sunlight, which can help to make treatment effects better detectable. In addition, it could be ethically more acceptable to investigate a new substance in a less severely affected subgroup of patients. However, the results of such trials are not generalisable to the entire patient population and must not be directly compared to results of studies that included a representative trial population.

#### Exclusion of patients with anemia

The phase II trials investigating bitopertin in EPP exclude patients with a blood hemoglobin concentration of less than 10 g/dL at screening. Because of its mode of action, i.e., decreased uptake of glycine (one of the substrates of the heme biosynthesis), bitopertin might decrease the blood hemoglobin concentration in EPP and thereby induce or worsen an already existing anemia (Fig. 1). In fact, previous trials investigating bitopertin in other conditions found a dose-dependent decrease in hemoglobin in their participants [62, 63]. While a minority, some patients with EPP, mostly females, have a hemoglobin of less than 10 g/dL. [17] Excluding patients with a severe anemia from the early phases of a drug development can be a reasonable precautionary measure. However, if not investigated in follow-up trials, safety and efficacy of bitopertin will remain unclear for this subgroup and might influence subsequent regulatory approval and treatment decisions.

#### Exclusion of patients with depression and suicidal ideations

For the bitopertin OLE study, patients with EPP are not accepted if they show signs of suicidal ideations or a certain degree of depression at screening. Rufener [11] in a qualitative interview study on psychosocial aspects of EPP reported that suicidal thoughts were common in the 12 investigated participants aged between seven to 46 years. In addition, a recent study on clinical characteristics of patients with EPP in the US cohort showed that 28% of the population has a diagnosis of depression in their clinical records (severity not reported) [25]. By excluding patients with suicidal ideations and/or depression, a severely affected and relevant subgroup of the patient population is excluded from gathering long-term data on bitopertin treatment in EPP. Moreover, suicidal ideations and depression are not listed as exclusion criteria in the phase II trials investigating bitopertin in EPP and the introduction of this exclusion criteria for the OLE raises questions about its safety, as discussed below.

### Efficacy outcomes

The primary endpoints used in the clinical trials investigating dersimelagon, bitopertin and cimetidine differ from each other, and from the endpoint used in the pivotal trial investigating afamelanotide in EPP. While some of the secondary endpoints assess the same aspects, the exact methods how the results were obtained need to be considered when comparing efficacy outcomes between the different interventions.

#### Efficacy of afamelanotide: time in sunlight without pain

For the pivotal trial investigating afamelanotide, time in sunlight without pain was recorded in 15-minute blocks in diaries over the 180 days trial period as the primary endpoint. As prespecified in the study protocol, only days on which the trial participants did not experienced any pain were included for the calculation, and only time spent in direct sunlight (and not, for example, time spent outdoors in the shade) [26]. Moreover, the patients were not asked to expose themselves to sunlight during the trial (see Section “Patient perspective: Selection of a less severely affected trial population”) but recorded the time they voluntarily spent in sunlight.

##### Measuring “pain” as an efficacy endpoint

The subjective nature of the endpoint, i.e., pain perception by the participants, has been discussed as a source for bias during the approval. [64] Being a symptomatic treatment, afamelanotide does not affect quantifiable biomarkers or objectively quantifiable disease signs in EPP. However, a “reduction in pain” is clinically meaningful and the extend of the provided benefit can be assessed by the patients themselves, as opposed to, for example, surrogate endpoints that measure change in biomarker expression [65].

#### Efficacy of dersimelagon: time to prodrome

The trials investigating dersimelagon use “time to prodrome” as their primary efficacy outcome measure. Prodromal symptoms are a newly introduced concept of early warning signals for a phototoxic reaction such as itching, tingling, burning and stinging. The protocol of the phase II study specifies that the patients “were encouraged to go outside during daylight hours long enough to induce prodromal sensations, but not trigger a phototoxic reaction” [52]. Moreover, the manual of the electronic sunlight exposure diary instructs the user to record “any time outdoors today between sunrise and sunset”, regardless of the weather conditions. Both specifications likely lead to an overestimation of the efficacy when compared to the afamelanotide trial results. Moreover, the concept of prodromi is not yet sufficiently established for EPP.

##### Prodromi in EPP: early warning signals or part of the phototoxic reaction?

In 2021, Wensink et al. [66] assessed the concept in an independent study in 89 patients with EPP from the US and Dutch cohort and defined a prodrome “as having two components that distinguish it from an excruciating pain attack: (1) the characteristic early warning symptoms on sun exposure and (2) the reversibility of the prodromal symptoms when patients immediately exit further sun exposure.” However, in their study, 76% of the participants reported having recovery times of one up to five days after experiencing a prodrome, and 46% experienced pain despite immediately exiting the sun. Some of the study participants even reported having pain as the first symptom they experience. Therefore, both requirements for a prodrome, i.e., being an early warning signal and being rapidly reversible, were not met in a considerable part of the investigated population.

##### Patient perspective: the concept of prodromi is not applicable to everyday life

In our experience the concept of prodromi is also not applicable to everyday life situations, in which the patients often cannot immediately retreat to a place without sunlight but need to travel to their workplaces etc [67]. As also artificial light can cause and/or worsen the symptoms, patients with EPP are not necessarily protected from further exacerbation of symptoms even if they immediately avoid further sunlight exposure [13, 15–18]. Consistent with our assessment, phototoxic pain events were observed in all treatment arms in the phase II trial investigating dersimelagon, i.e., in the placebo, low dose and high dose treatment groups [52]. Therefore, the endpoint “time to prodrome” does not prevent participants from developing severe reactions.

#### Efficacy of bitopertin and cimetidine: decrease in PPIX

**Fig. 5:**
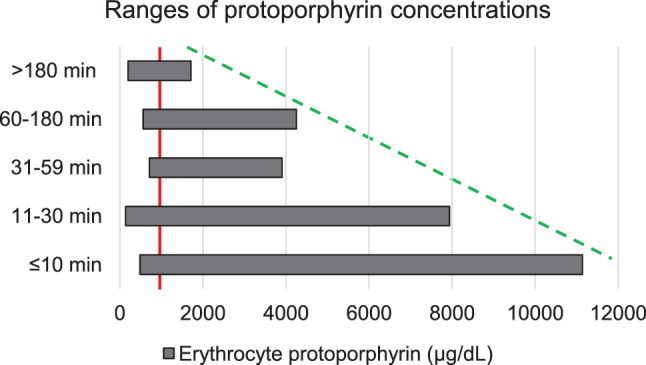
Ranges of PPIX concentrations of patients with different time to onset to symptoms, US cohort (data adopted from [18]): while high blood PPIX concentrations positively correlate with faster onset of symptoms (green dotted line), a blood PPIX concentration in the middle and lower ranges does not predict time to onset of symptoms. For example, a blood PPIX concentration of 1000 μg/dL can be associated with a time to onset of symptoms between less than 10 minutes up to more than 3 hours (red solid line)

Investigating potentially causative treatment options, the clinical trials for bitopertin and cimetidine use the surrogate endpoint “decrease in PPIX” as their primary efficacy endpoint. PPIX is well established as the cause for the phototoxic reactions in EPP and blood PPIX concentrations are objectively quantifiable. However, blood PPIX concentrations do not predict how clinically meaningful a treatment is: In the same study on the US patient cohort by Balwani et al. [18] as discussed above, higher blood PPIX concentrations did in fact positively correlate with faster onset of symptoms (Fig. 4, dotted green line) but PPIX concentration in the middle and lower ranges did not correlate at all. (Fig. 5, solid red line) Therefore, blood PPIX concentration alone cannot be used to demonstrate a meaningful effect but needs to be supplemented with clinically relevant outcome measures (as is the case in the clinical trial protocols for both, bitopertin and cimetidine, for details, see table S4). Importantly, blood PPIX concentrations also cannot be used to stratify EPP trial populations regarding disease severity and, vice versa, do not demonstrate a representative inclusion of trial participants.

##### No evidence for benefit in patients with liver disease is generated

One of the expected benefits of causative treatments in EPP is their potential to reduce PPIX-induced liver damage [35]. However, in the currently conducted clinical trials investigating the causative treatment options bitopertin and cimetidine, patients with clinically significant liver dysfunction are excluded from participation. Therefore, there currently is no evidence generated for the potential beneficial effects of bitopertin and cimetidine for this subgroup.

### Quality of life and patient reported outcome measures:

According to the clinical trial protocols, different generic and disease-specific instruments to quantify Quality of Life and Patient Reported Outcome Measures are used in the clinical trials investigating pharmacotherapies in EPP (Table 1). The phase II trials investigating dersimelagon and cimetidine both use the Patient-Reported Outcomes Measurement Information System 57 (PROMIS-57), and the Patient Global Impression of Change (PGIC) scale is used in trials investigating dersimelagon and bitopertin. According to the published protocols, only afamelanotide has been assessed with a disease specific tool, the EPP-QoL [68].

### Safety aspects

While we did not systematically assess safety outcomes in our analysis, we investigated aspects relevant to EPP when comparing the four investigated pharmacotherapies.

#### Comparing adverse events of a slow-release implant formulation with oral formulations

Afamelanotide is administered every two months as a slow-release implant formulation. In contrast, all three currently investigated new pharmacotherapies are oral formulations that are taken daily. Therefore, to meaningfully compare their safety with that of afamelanotide, adverse reactions need to be reported in detail [49, 50]. For example, it should not only be reported whether a participant experienced nausea during the trial, but how often the nausea occurred and how severe it was, when it occurred (e.g., time since drug administration) and for how long it lasted.

#### Afamelanotide and dersimelagon: comparable mode of action should lead to comparable safety measures

Afamelanotide and dersimelagon both bind to the melanocortin 1 receptor (MC1R) on melanocytes. Therefore, it can be assumed that comparable safety measures as mandated for afamelanotide should be applicable for dersimelagon, too. Although based on inconclusive evidence, it has been suggested that activation of the MC1R can promote the development of melanoma. EPP is not associated with a known increased risk for any skin cancers [53]. Still, the clinical trials investigating afamelanotide and dersimelagon exclude patients having any precancerous skin lesions and/or skin cancer or melanoma at baseline. According to the EMA, at the time of the approval of afamelanotide in 2014, no signal for an increased cancer risk had been detected in any of the clinical trials and long-term observational studies from compassionate use and expanded access programmes in patients with EPP under treatment with afamelanotide since the first phase II trial in 2006 [69]. Nevertheless, as a condition for regulatory approval, the EMA had requested an extensive PASS that, amongst other measures, requires the patients to undergo a full body photography and screening for precancerous skin lesions and melanoma every six months [29, 32]. In addition, according to EMA`s Summary of Product Characteristics (SmPC) document, patients with EPP “can be expected to significantly increase their exposure to sunlight and UV light while on treatment with afamelanotide”. [67] Therefore, the SmPC recommends use of UV protection measures to reduce the risk for UV-induced skin damage while under treatment with afamelanotide [69]. The data collected in the PASS is evaluated annually by the EMA and a positive risk-benefit assessment is a condition for the continuation of the marketing authorisation. Although the EMA until now (May 2025) every year issued a positive recommendation, the PASS and strict measures against off-label use need to run indefinitely. The feasibility of applying some of the measures of the PASS to dersimelagon, for example the prevention of off-label use, would need to be considered.

#### Repurposed drugs: safety data generated in conditions other than EPP

Drugs which have been previously assessed in other conditions have the advantage of an existing set of safety data. While drug repurposing has been successfully conducted in several diseases, including rare diseases, disease-specific aspects still need to be carefully considered [70, 71].

##### Repurposing of bitopertin

Bitopertin has an extensive body of safety data from clinical trials in healthy volunteers and patients with other conditions, with collectively around 4000 participants [45]. For EPP, safety data is collected in controlled clinical trials since 2022 and the ongoing OLE. Being a systemic treatment, off-target effects of bitopertin on the heme metabolism in other cell types than erythrocytes cannot be excluded for EPP1, in which the pathogenic variant in the *FECH* gene is present in all cells [2]: The target of bitopertin, i.e., glycine transporter 1, is for example also expressed in glial cells in the brain [72]. Moreover, the reason for excluding patients with EPP having suicidal ideations and/or a certain degree of depression from the bitopertin OLE study needs to be explained. According to the trial protocols on clinicaltrials.gov, depression and suicidality are also exclusion criteria of trials investigating bitopertin in other haematological disorders, i.e., beta-thalassemia (NCT03271541) and Diamond-Blackfan anemia (NCT05828108), which hints towards a suspected association. In fact, in a previous trial investigating bitopertin in schizophrenia (NCT01235559), one patient committed suicide, which was assessed as “related to study drug” [73].

##### Repurposing of cimetidine

Cimetidine has been approved in the mid 1970ties for short-term treatment of gastroesophageal reflux disease, heartburn and duodenal and gastric ulcers [56]. In 2016, Tu et al. [55] suggested cimetidine as a causative treatment option for EPP. A recently published *in-vitro* study did not find evidence for the hypothetical mode of action, i.e., the inhibition of the enzymatic activity of ALAS2, the first and rate limiting enzyme of the erythropoietic heme biosynthesis. [74] However, an uncontrolled study previously conducted in one academic centre in Denmark in 18 adult patients with EPP1 receiving up to 1600 mg cimetidine daily for up to two years reported a median 20% reduction in blood PPIX concentrations [75]. Known adverse effects of cimetidine include reduced uptake of dietary iron [76]. Iron is not only a substrate but also a regulator of the erythroid heme biosynthesis and a decrease in iron availability has been shown to reduce blood PPIX and hemoglobin concentrations in EPP1 [6, 43]. As most patients in the Danish study showed a decrease in their blood hemoglobin concentration, the observed decrease in PPIX might be explained by a decrease in dietary iron uptake under cimetidine treatment. If confirmed as the mode of action of cimetidine in EPP1, anemia needs to monitored and alternative methods to reduce iron availability that are also suitable for long-term use such as phlebotomies should be considered. Moreover, in XLEPP (and likely EPP2), a reduction in iron availability by cimetidine might have detrimental effects: because of differences in its pathophysiology as compared to EPP1, iron supplementation is currently used to decrease blood PPIX concentration in XLEPP [46].

### Identified ethical issues

Besides the methodological problems we identified ethically challenging aspects in the clinical trial protocols of the currently investigated pharmacotherapies. [67]

#### Measuring treatment effects against a placebo-control arm

The use of placebo-control groups in clinical trials has been widely criticised by patient organisations [77–79]. According to the provisions of the 2024 version of the Declaration of Helsinki, the use of a placebo-control arm in trials investigating new treatment options in conditions for which a treatment exists is indeed only acceptable in case of “compelling and scientifically sound methodological justifications”. [80] For EPP, the EMA during their evaluation of afamelanotide for regulatory approval had concluded that “participation in a [placebo-]controlled clinical trial would expose patients to a risk of severe phototoxicity and pain that would not be ethically acceptable” [69]. Yet, all new pharmacotherapies for EPP are currently investigated in placebo-controlled clinical trials, some of which even include adolescents [67]. Moreover, patients participating in the clinical trials investigating dersimelagon and cimetidine must agree to expose to sunlight until symptoms develop (see Section “Patient perspective: Selection of a less severely affected trial population”). Potential solutions such as alternative and adaptive trial designs and examples of trial designs in other ultra-rare diseases are discussed in section “Suggestions for improved trial designs in EPP”.

#### “In-clinic sunlight exposure test” in patients who have an anxiety to be exposed to light

In addition, according to the full trial protocol published together with the results of the phase II study investigating dersimelagon, the participants were subjected to an “in-clinic sunlight exposure test”, in which their tolerance to sunlight was assessed at baseline and at the end of the study, at week 16: “Site staff will lead subjects into a setting with sun-light exposure and subjects will be provided with a timer (e.g. near window, outdoors) to collect the period of time it takes for the subject to experience the onset of the first prodromal symptom” [52]. Given the anxiety of the patients to expose themselves to light, the inadequate nature of prodromi as an endpoint (see Section “Patient perspective: Selection of a less severely affected trial population”) and the lack of options to treat the potentially inflicted severe pain that is exacerbated by environmental factors such as artificial light, wind and heat during and after the visit, in-clinic sunlight exposure tests in patients with EPP are ethically problematic. From a patient perspective, we even consider them ethically unacceptable.

### Suggestions for improved trial designs in EPP

In our assessment, some of the identified ethical and methodological issues associated with placebo-controlled trials (see Section “Patient perspective: Selection of a less severely affected trial population”) could be addressed by alternative trial designs.

#### Head-to-head studies between afamelanotide and dersimelagon

In double-blind randomised trials, neither the participant nor the investigator is supposed to have knowledge about the treatment allocation, as this potentially biases the trial results [81]. However, afamelanotide and dersimelagon both increase skin pigmentation and have been shown to lead to partial unblinding of the trials (Fig. 1): As discussed in the German HTA report, 19% of the trial participants in the treatment arm of the pivotal study investigating afamelanotide noticed a discoloration of their skin at the implantation site which in these patients likely caused unblinding. [82] Likewise, in the phase II trial investigating dersimelagon, 97% of the patients in the low dose group, 100% of the patients in the high dose group and 59% of the patients in the placebo group correctly guessed their treatment allocation [52]. During the approval procedures of afamelanotide, the EMA assessed that the lack of complete blinding of the trials impaired the validity of the evidence for efficacy, but also acknowledged that there was no possibility of blinding the studies appropriately [69]. In contrast, dersimelagon could now be investigated in a double-blind manner if all trial participants receive both, a pill and an implant, with one being an active and the other a sham treatment: In such a trial, any noticeable skin pigmentation could be caused by either one of the substances. Moreover, such head-to-head studies would also avoid the ethical issues associated with placebo-control groups and would enhance comparability of the trial results. Head-to-head studies have been successfully conducted in other ultra-rare and heterogenous diseases: One example is the comparison of the safety and efficacy of the enzyme replacement therapy imiglucerase (an infusion) with the orally administered eliglustat for treating type 1 Gaucher disease [83].

#### Investigating bitopertin as an adjunct therapy

As bitopertin might not be suitable for treating patients with a low hemoglobin and/or suicidal ideations or depression, relevant and severely affected parts of the patient population might not benefit from its development. Instead of investigating bitopertin as an individual treatment option, it could be assessed in combination with afamelanotide for patients at risk of developing EPP-related liver disease. An example for a successful development of an adjunct pharmacotherapy in an ultra-rare disease is arimoclomol for treating patients suffering from Niemann-Pick disease type C. Arimoclomol has been recently approved by the FDA as a combination therapy with miglustat, the existing treatment option. In the pivotal clinical trial, patients with Nieman-Pick disease type C already under therapy with miglustat continued with the treatment and were stratified to the different treatment arms receiving or not receiving arimoclomol. [84] Alternatively, innovative approaches such as master protocol trials which assess several drugs for the same disease and/or one drug in several subgroups of a disease, or alternative and adaptive trial designs with an initial placebo phase followed by an early escape to active comparator could be potential solutions, too [85, 86].

### Ultra-rare diseases: new approvals might not result in more treatment options

It could be argued that additional treatments, even if they have certain limitations such as not being suitable for some disease subgroups, might lead to more choice for the entire EPP patient population. However, as shown by an analysis commissioned by the US National Organization for Rare Disorders (NORD), new approvals in the field of ultra-rare diseases might not necessarily result in more treatment options but instead can trigger the discontinuation of existing drugs: because of the very small market size, an existing drug might become unprofitable and therefore discontinued if a new one becomes available [87]. If the effects of new drugs are not assessed in a manner that allow a comparison with the existing drug already before their approval, there is a risk that patients might be left with treatments that are less safe and effective.

### Limitations and strengths of the study

A major limitation of our study is that the trials included in the analysis are from different stages of the clinical development, i.e., phase II, III and IV: Phase II trials might not necessarily aim to quantify efficacy but rather try to answer questions such as dose-finding or demonstrate a proof-of-concept. Ideally, outcomes of trials should be compared between trials from the same phase. We nevertheless deemed it worthwhile to already now conduct the presented analysis, because the finding might support more patient-centred and clinically relevant trial designs in EPP.

Another issue is that some of the trial protocols as published in the registries differ from information obtained from other sources. Incomplete and inconsistent reporting of clinical trial protocols and outcomes is a known issue that may introduce bias and render research based on such information unreliable [88, 89]. For example, it is not clear how and why patients below age 18 have been recruited for the bitopertin trial. Trial registries are supposed to be a trusted source of information for the public. If basic information such as included age groups differ between the published trial protocol and information provided in, for example, conference abstracts, the question can be raised on how reliable the information on other aspects (adverse events, efficacy results) is reflected in the registries [89].

A further limitation of the manuscript is that for our analysis, only patients who are members of the IPPN have contributed their patient perspective on EPP-related aspects. Therefore, the opinions shared in the article should be confirmed in larger and more heterogenous patient groups. However, a strength of the study is that the IPPN is a financially independent patient organisation, and the primary interest of its members are treatments that are safe, effective and accessible to every patient. Its members have specific knowledge regarding drug development, approval and reimbursement procedures and have lived experiences with the disease and one or more investigational therapies, some even as participants in clinical trials and research studies.

Moreover, the presented study is the first analysis comparing trial protocols of currently investigated pharmacotherapies for treating EPP in detail. During the analysis, several aspects to be considered for methodically and ethically sounder trial designs for the further development of newly investigated pharmacotherapies were identified and potential solutions are discussed in the presented manuscript.

## Conclusion

For patients, and in the interest of a scientifically robust drug development in general, it is important that current trial designs and efficacy endpoints are built on the experiences and learnings of previous trials with the aim to increase the scientific knowledge while at the same time reduce the risks and burdens of participation [78–80]. Moreover, to promote access after regulatory approval, trials need to be designed in a manner that supports reimbursement decisions, by, for example, limiting potential sources for bias (such as unblinding of the participants) and uncertainties regarding their relative efficacies and safeties [90]. Our study resulted in the identification of four drugs that are currently investigated for treating EPP, i.e., afamelanotide, dersimelagon, bitopertin and cimetidine. However, the detailed analysis of the trial protocols retrieved from publicly accessible trial registries demonstrates that with the currently used trials designs, efficacy and safety of the new therapies are assessed in a manner that does not allow to compare the results with those of afamelanotide, the approved treatment, and that the patients are unnecessarily burdened when participating in these trials: All four pharmacotherapies were/are assessed in clinical trials against a placebo-control group or against baseline, which means that their relative efficacies and safeties remains unknown. In addition, our study revealed considerable differences in the included trial populations and outcome measures between the trials, which makes comparisons of the results unfeasible [58]. For example, one cannot directly compare the time spent in direct sunlight with the time generally spent outdoors, irrespective of the weather conditions. However, in ultra-rare diseases, understanding the potential benefits and risks of new treatments and how they compare to existing treatments before their potential approval is crucial: because of the very small market size, new drugs for ultra-rare diseases might not result in more treatment options but may cause the existing drug to disappear because it might no longer be profitable [87]. We are also concerned about ethically challenging aspects identified in some of the trial protocols such as using placebo control groups despite the Declaration of Helsinki requires that new drugs are tested against the existing treatment option, and the expectation that the trial participants expose to sunlight until symptoms develop despite the known anxiety of the patients and the potentially inflicted severe and untreatable pain [69, 80]: Principal Investigators, Ethical Review Boards, regulatory authorities and the sponsors of trials should be aware that especially in case of lack of (access to) efficacious treatments, patients might be more willing to accept burdensome conditions for clinical trials despite ethically challenging aspects. [91] We wrote this manuscript because we are convinced that improving the design of trials assessing new pharmacotherapies for EPP is necessary and possible. Moreover, it is encouraging that most of the identified issues are not specific for EPP, and that the value of including the patient perspective in clinical trials is increasingly recognized and hopefully soon will become a standard requirement for regulatory approval of new medicines [91–95].

## Electronic supplementary material

Below is the link to the electronic supplementary material.

Supplementary Material 1

Supplementary Material 2

## Data Availability

All data and materials are publicly available

## Abbreviation

ALA: Delta-aminolevulinic acid
ALAS2: Delta-aminolevulinate synthase 2
ALAD: Delta-aminolevulinate dehydratase
C-SSRS: Columbia Suicide Severity Rating Scale
Copro`gen III: Coproporphyrinogen III
CPLX: Caseinolytic mitochondrial matrix peptidase chaperone subunit
DLQI: Dermatology Life Quliyt Index
EMA: European Medicines Agency
EPP: Erythropoietic protoporphyrias
EPP1: Erythropoietic protoporphyria 1
EPP2: Erythropoietic protoporphyria 2
EPP-QoL: EPP-Quality of Life
eSED: Electronic sunlight exposure diary
FECH: Ferrochelatase
FDA: Food and Drug Administration
HMBS: Hydroxymethylbilane synthase
HMB: Hydroxymethylbilane
HTA: Health technology assessment
IRDiRC: International Rare Diseases Research Consortium
IPPN: International Porphyria Patient Network
MC1R: Melanocortin 1 receptor
OLE: Open label extension study
OMIM: Online Mendelian Inheritance in Man
PASS: Post-authorisation safety and effectiveness study
PBG: Porphobilinogen
PHQ-8: Patient Health Questionnaire
PROMIS-57: Patient-Reported Outcomes Measurement Information System 57
PK: Pharmacokinetics
PPIX: Protoporphyrin
PPOX: Protoporphyrinogen oxidase
Proto`gen IX: Protoporphyrinogen IX
RCT: Randomised, placebo-controlled clinical trial
SmPC: Summary of Product Characteristics
TGA: Therapeutic Goods Administration
UROD: Uroporphyrinogen decarboxylase
Uro`gen III: Uroporphyrinogen III
UROS: Uroporphyrinogen III synthase
XLEPP: X-linked erythropoietic protoporphyria
