# Supplementary material for: New pharmacotherapies for the erythropoietic protoporphyrias: an analysis of trial protocols from a patient perspective
Source: Orphanet J Rare Dis. 2025 Dec 29;20:637. doi: 10.1186/s13023-025-04170-9 (PMC12751534; doi:10.1186/s13023-025-04170-9)
Supplement: Supplementary file 1 — Supplementary Material 1 [file 13023_2025_4170_MOESM1_ESM.pdf]

Supplement 1, Table S1-S3

New pharmacotherapies for the erythropoietic protoporphyrias. An analysis of the trial protocols from a patient perspective

Dechant et al.

Table S1: Registries screened for clinical trials and studies involving patients with EPP p.3

Table S2: Clinical trials in erythropoietic protoporphyria

Clinical trials identified in the public trial registries. No hits were retrieved for the registries: Asia Pacific Clinical Trials; Pan African Clinical Trials Registry; WHO International Clinical Trials Registry Platform.

Table S2a: Clinicaltrials.gov p. 4-7
Table S2b: EudraCT p. 8-9
Table S2c: Clinical Trials Information System (CTIS) p. 10
Table S2d: Swiss National Clinical Trials Portal p. 11
Table S2e: Australian New Zealand Clinical Trial Registry p. 12-13
Table S2f: Japan Registry of Clinical Trials (JRCT) p. 14-15
Table S2g: Orphanet directory of ongoing research projects, clinical trials and biobanks p. 16

Supplement 1: New pharmacotherapies for the erythropoietic protoporphyrias. An analysis of the trial protocols from a patient perspective; Dechant et al.

Table S3: Clinical trial protocols of pharmacotherapies currently investigated in erythropoietic protoporphyria

Table S3a: Afamelanotide	p. 17-46
Table S3b: Dersimelagon	p. 47-59
Table S3c: Bitopertin	p. 60-68
Table S3d: Cimetidine	p. 69-70

Abbreviations:

PICO	Population/Intervention/Comparator/Outcome
OL	Open label
OLE	Open label extension
PASS	Post-authorisation safety and effectiveness study

Colour code:

Table S2: Currently ongoing trials are marked in green.

Table S3:

P = Population, blue

I = Intervention, pink

C = Comparator, grey

O = Outcome, green

Safety: yellow

Table S1: Registries screened for clinical trials and studies involving patients with EPP

Registry	Homepage	Country/ Region
ClinicalTrials.gov	https://clinicaltrials.gov/	USA
EudraCT	https://eudract.ema.europa.eu/	EU
Clinical Trials Information System (CTIS)	https://euclinicaltrials.eu/	EU
Swiss National Clinical Trials Portal	https://www.humanforschung-schweiz.ch/en/trial-search/	Switzerland
Australian New Zealand Clinical Trial Registry	https://www.anzctr.org.au/	Australia, New Zealand
Japan Registry of Clinical Trials	https://jrct.niph.go.jp/en-top	Japan
Asia Pacific Clinical Trials	https://en.cmicgroup.com/solutions/specialized-services/asia-pacific-clinical-trials/	Asia-Pacific
Pan African Clinical Trials Registry	http://www.edctp.org/pan-african-clinical-trials-registry/	Africa
WHO International Clinical Trials Registry Platform	https://www.who.int/clinical-trials-registry-platform	International
Orphanet directory of ongoing research projects, clinical trials and biobanks	https://www.orpha.net	International

Table S2a: Clinical trials in the erythropoietic protoporphyrias listed on clinicaltrials.gov

Last accessed 20 February 2025

	Study ID Number	Other Study IDs	Titel	Intervention	Start date	Completion date/status
1	NCT06388642	CUV052	Pharmacokinetics of Afamelanotide in EPP Patients	Afamelanotide 16mg implant	2024-03-07	Recruiting
2	NCT06144840	MT-7117-A-302<br><br>jRCT2031230656 (Registry Identifier) (REGISTRY: Japan Registry of Clinical Trials (jRCT))<br><br>2023-506735-15-00 (EU Trial (CTIS) Number)	INcreased Sun Exposure Without Pain In Research Participants With EPP or XLP (INSPIRE)	Dersimelagon	2023-12-11	Recruiting
3	NCT05883748	DISC-1459-501	Open-Label, Long-Term Extension Study to Investigate the Safety, Tolerability, and Efficacy of DISC-1459 (Bitopertin) in Participants With EPP	Bitopertin	2023-08-31	Enrolling by invitation
4	NCT05780840	Dihydroxyacetone	Protection Against Visible Light by Dihydroxyacetone in Erythropoietic Protoporphyria	Dihydroxyacetone	2023-02-23	Recruiting
5	NCT05572125	Iron	Iron Therapy in Erythropoietic Protoporphyria	Oral iron	2022-10-17	Completed
6	NCT05308472	DISC-1459-201	Study of Bitopertin to Evaluate the Safety, Tolerability, Efficacy, and PPIX Concentrations in Participants With EPP	Bitopertin	2022-10-31	Completed

7	NCT05020184	2021P002095<br><br>PC7211 (Other Identifier) (OTHER: Porphyrias Consortium Protocol Number)<br><br>1R01FD007287-01 (U.S. FDA Grant/Contract)	Effect of Oral Cimetidine in the Protoporphyrias	Cimetidine	2022-06-14	Active, not recruiting
8	NCT05005975	MT-7117-A-301<br><br>jRCT2041210146 (Registry Identifier) (REGISTRY: Japan Registry of Clinical Trials (jRCT))<br><br>2021-001831-17 (EudraCT Number)	Extension Study to Evaluate Safety and Tolerability of Oral Dersimelagon (MT-7117) in Subjects With Erythropoietic Protoporphyria (EPP) or X-Linked Protoporphyria (XLP)	Dersimelagon	2021-08-10	Recruiting
9	NCT04578496	CUV037	A Safety Extension Study in Patients With Erythropoietic Protoporphyria (EPP)	Afamelanotide	2011-07-11	Completed, 2014-02-27
10	NCT04402489	MT-7117-G01	Study to Evaluate Efficacy, Safety, and Tolerability of MT-7117 in Subjects With Erythropoietic Protoporphyria or X-Linked Protoporphyria	Dersimelagon	2020-06-01	Completed, 2022-07-26
11	NCT04053270	CUV017	Multicentre Phase III Erythropoietic Protoporphyria Study	Afamelanotide	2007-05	Completed, 2009-12-09
12	NCT03682731	Erythropoietic protoporphyria	Light Exposure Patterns and Symptoms Among Patients With Erythropoietic Protoporphyria	Observational	2017-04-01	Completed, 2017-08-01
13	NCT03520036	MT-7117-A01	Study to Evaluate Efficacy, Safety, and Tolerability of MT-	Dersimelagon	2018-07-05	Completed, 2019-09-28

			7117 in Subjects With Erythropoietic Protoporphyria			
14	NCT02979249	GCO 08-0959-1001<br><br>U54DK083909 (U.S. NIH Grant/Contract)	Oral Iron for Erythropoietic Protoporphyrias (EPP)	Oral Iron	2016-12	Completed, 2019-07-19
15	NCT01880983	7202	Mitoferrin-1 Expression in Erythropoietic Protoporphyria (Porphyria Rare Disease Clinical Research Consortium (RDCRC))	Observational	2011-11	Completed, 2020-12-31
16	NCT01688895	GCO 08-0959-04<br><br>HSM12-00307 (Other Identifier) (OTHER: Mount Sinai School of Medicine)<br><br>U54DK083909 (U.S. NIH Grant/Contract)	Erythropoietic Protoporphyrias: Studies of the Natural History, Genotype-Phenotype Correlations, and Psychosocial Impact	Observational	2012-07	Completed, 2019-07-01
17	NCT01605136	CUV039	Phase III Confirmatory Study in Erythropoietic Protoporphyria	Afamelanotide	2012-05	Completed, 2013-07
18	NCT01561157	GCO 10-1102	Longitudinal Study of the Porphyrias	Observational	2010-11-01	Recruiting
19	NCT01550705	UTINH<br><br>U54DK083909 (U.S. NIH Grant/Contract)	Effect of Isoniazid on Protoporphyrin Levels in Erythropoietic Protoporphyria (INHEPP)	Isoniazid	2012-03	Terminated, 2015-12
20	NCT01422915	2010P002253	Sorbent Therapy of the Cutaneous Porphyrias (EPP)	Colestipol	2011-05	Completed, 2012-03
21	NCT01097044	CUV030	Phase II Confirmatory Study in Erythropoietic Protoporphyria (EPP)	Afamelanotide	2010-04	Completed, 2011-04
22	NCT00979745	CUV029	Phase III Confirmatory Study in Erythropoietic Protoporphyria (EPP)	Afamelanotide	2009-09	Completed, 2011-05

23	NCT00206869	KF 01-169/02	Does Exercise and Heat Increase the Lightsensibility in Patients With Erythropoietic Protoporphyria	Behavioral: exercise, Behavioral:heat, Procedure:red light illumination	2003-01	Unknown status
24	NCT00004831	199/13413<br><br>BWH-FDR000996-EF<br><br>SLRH-CU-FDR000996-EF	Study of Cysteine Hydrochloride for Erythropoietic Protoporphyria	Cysteine hydrochloride	1996-10	Completed, 1998-07
25	NCT00004940	199/13376<br><br>BWH-FDR000996-DR	Phase III Study of L-Cysteine in Patients With Erythropoietic Protoporphyria	Cysteine hydrochloride	1996-05	Completed, 2001-09

Currently ongoing trials are marked in green.

Table S2b: Clinical trials in the erythropoietic protoporphyrias listed on clinicaltrialsregister.eu (EudraCT)

Last accessed: 20 February 2025

	**Study ID, EudraCT Number**	**Other study IDs**	**Titel**	**Intervention**	**Start date**	**Completion date/ status**
1	2007-002863-28	n.a.	Investigation into the use of colestyramine as a therapy for patients with erythropoietic protoporphyria	Colestyramine	26 Sep 2007	3 Nov. 2011 Completed
2	2021-001831-17	= MT-7117-A-301	A Phase 3, Multicenter, Open-label, Long-term, Extension Study to Evaluate Safety and Tolerability of Oral Dersimelagon (MT-7117) in Subjects with Erythropoietic Protoporphyria (EPP) or X-Linked Pr..	Dersimelagon	7 April 2022	n.a.
3	2009-011018-51	= CUV029	Phase III, Multicentre, Double-Blind, Randomised, Placebo-Controlled Study to Confirm the Safety and Efficacy of Subcutaneous Bioresorbable Afamelanotide Implants in Patients with Erythropoietic …	Afamelanotide	6 Aug 2009	09 May 2011
4	2019-004226-16	= MT-7117-G01	A Phase 3, Multicenter, Randomized, Double-Blind, Placebo-Controlled Study to Evaluate Efficacy, Safety, and Tolerability of MT-7117 in Adults and Adolescents with Erythropoietic Protoporphyria or ...	Dersimelagon	n.a.	26 July 2022

5	2007-000636-13	=CUV017	A Phase III, Multicentre, Randomised, Placebo-Controlled Study to Evaluate the Safety and Efficacy of Subcutaneous Bioresorbable CUV1647 Implants in Patients with Erythropoietic Protoporphyria (EPP)	Afamelanotide	17 June 2008	

Currently ongoing trials are marked in green.

Table S2c: Clinical trials in the erythropoietic protoporphyrias listed in CTIS (EU Clinical Trials Information System)

Last accessed: 20 February 2025

	Study ID, EUCT (CTIS) Number	Other study IDs	Titel	Intervention	Start date	Completion date/ status
1	EUDRA CT : 2021-001831-17	MT-7117-A-301<br><br>=NCT05005975	A Phase 3, Multicenter, Open-label, Long-term, Extension Study to Evaluate Safety and Tolerability of Oral Dersimelagon (MT-7117) in Subjects with Erythropoietic Protoporphyria (EPP) or X-Linked Protoporphyria (XLP)	Dersimelagon	19 April 2022	Ongoing
2	EUCT number: 2023-506735-15-00	=MT-7117-A-302	MT-7117-A-302 Study: A Phase 3, Multicenter, Randomized, Double-Blind, Placebo-Controlled Study to Evaluate Efficacy, Safety, and Tolerability of MT-7117 in Adults and Adolescents with Erythropoietic Protoporphyria or X-Linked Protoporphyria	Dersimelagon	05 April 2024	Ongoing
3	2023-507311-35-00	= NCT06388642<br><br>=CUV052	A Study to Evaluate the Pharmacokinetics of Afamelanotide in Patients with Erythropoietic Protoporphyria (EPP).	Afamelanotide	19 Feb. 2024	Ongoing

Currently ongoing trials are marked in green.

Table S2d: Clinical trials in the erythropoietic protoporphyrias listed in the Swiss National Clinical Trials Portal

Last accessed: 14 February 2025

	Study ID, Swiss National Clinical Trials Portal number	Other study IDs	Titel	Intervention	Start date	Completion date/ status
1	HumRes64863<br>SNCTP000005837<br>BASEC2023-02180	= CUV052<br><br>= EUCTR2023-507311-35	A study to evaluate the pharmacokinetics of Afamelanotide in patients with erythropoietic protoporphyria (EPP).	SCENESSE® (Afamelanotide 16 mg implant)	12.03.2024 (date of authorisation)	Unknown
2	HumRes65456	= CTIS2023-507311-35-00	A Study to Evaluate the Pharmacokinetics of Afamelanotide in Patients with Erythropoietic Protoporphyria (EPP)	SCENESSE 16 mg implant	n.a.	Recruitment completed
3	HumRes6296	= CUV010, ACTRN12606000535572	Phase II Study of CUV1647 in Erythropoietic Protoporphyria (EPP)	CUV1647 (20 mg implant) = afamelanotide	n.a.	Recruitment completed

Currently ongoing trials are marked in green.

Table S2e: Clinical trials in the erythropoietic protoporphyrias listed in the Australian New Zealand Clinical Trial Registry

Last accessed: 20 February 2025

	Study ID, ANTCTR Number	Other study IDs	Titel	Intervention	Start date	Completion date/ status
1	ACTRN12622000799752	DISC-1459-202	A Phase 2, Randomized, Open Label Study of Bitopertin to Evaluate the Safety, Tolerability, Efficacy, and Protoporphyrin IX (PPIX) Concentrations in Participants with Erythropoietic Protoporphyria (EPP)	Bitopertin	01 July 2022	Recruiting
2	ACTRN12607000261415	n.a.<br><br>(2007-000636-13<br><br>CUV017)	A Phase III, Multicentre, Randomised, Placebo-Controlled Study to Evaluate the Safety and Efficacy of Subcutaneous Bioresorbable CUV1647 Implants in Patients with Erythropoietic Protoporphyria (EPP)	Afamelanotide	01 June 2007	(Active, not recruiting) Not up to date, last updated 8/5/2009
3	ACTRN12606000535572	=CUV010	A Multicentre, Phase II, Open Label Study to Evaluate the Safety of CUV1647 and to Evaluate the Effect of Subcutaneous Implants of CUV1647 on the Time to Artificially Provoked Symptoms in Patients with Erythropoietic Protoporphyria (EPP)	Afamelanotide	01 September 2006	Stopped early

4	NCT06144840*	jRCT2031230656<br><br>MT-7117-A-302	INcreased Sun Exposure Without Pain In Research Participants With EPP or XLP	Dersimelagon	11/12/2023	Recruiting
5	NCT05883748*	DISC-1459-501	Open-Label, Long-Term Extension Study to Investigate the Safety, Tolerability, and Efficacy of DISC-1459 (Bitopertin) in Participants With EPP	Bitopertin	31/08/2023	Recruiting
6	NCT05005975*	jRCT2041210146<br><br>MT-7117-A-301	Extension Study to Evaluate Safety and Tolerability of Oral Dersimelagon (MT-7117) in Subjects With Erythropoietic Protoporphyria (EPP) or X-Linked Protoporphyria (XLP)	Dersimelagon	10/08/2021	Recruiting
7	NCT04402489*	MT-7117-G01	Study to Evaluate Efficacy, Safety, and Tolerability of MT-7117 in Subjects With Erythropoietic Protoporphyria or X-Linked Protoporphyria	Dersimelagon	1/06/2020	Completed, 26/07/2022

Currently ongoing trials are marked in green.

*Full trial details at clinicaltrials.gov

Table S2f: Clinical trials in the erythropoietic protoporphyrias listed on Japan Registry of Clinical Trials (jRCT)

Last accessed 20 February 2025

	Study ID Number	Other Study IDs	Titel	Intervention	Start date	Completion date/status
1	jRCT2031230656	NCT06144840<br><br>MT-7117-A-302<br><br>CTIS: 2023-506735-15-00	A Phase 3, Multicenter, Randomized, Double-Blind, Placebo-Controlled Study to Evaluate Efficacy, Safety, and Tolerability of MT-7117 in Adults and Adolescents with Erythropoietic Protoporphyria or X-Linked Protoporphyria/<br><br>A Study to Evaluate Efficacy, Safety, and Tolerability of MT-7117 in Subjects with Erythropoietic Protoporphyria or X-Linked Protoporphyria (INcreased Sun exposure without Pain In Research participants with EPP or XLP) (INSPIRE)	Dersimelagon	April. 01, 2024	Recruiting
2	jRCT2041210146	NCT05005975<br><br>MT-7117-A-301<br><br>CITIS: 2024-514466-38-00	A Phase 3, Multicenter, Open-label, Long-term, Extension Study to Evaluate Safety and Tolerability of Oral Dersimelagon (MT-7117) in Subjects with Erythropoietic Protoporphyria (EPP) or X-Linked Protoporphyria (XLP)/<br><br>Extension Study to Evaluate Safety and Tolerability of Oral Dersimelagon (MT-7117) in	Dersimelagon	May. 06, 2022	Recruiting

			Subjects With Erythropoietic Protoporphyria (EPP) or X-Linked Protoporphyria (XLP)			

Currently ongoing trials are marked in green.

Table S2g: Clinical trials in the erythropoietic protoporphyrias listed in the Orphanet, Directory of ongoing research projects, clinical trials and biobanks

Last accessed: 20 February 2025

	Study ID, Orphanet number	Other study IDs	Titel	Intervention	Start date	Completion date/ status
1	n.a.<br><br>https://www.orpha.net/de/research-trials/research-projects/597235?name=&mode=&country=	n.a.	SCENESSE® PASS-001: A Post-Authorisation Disease Registry Safety Study to Generate Data on the Long-Term Safety and Clinical Effectiveness of SCENESSE® (Afamelanotide 16mg) in Patients with Erythropoietic Protoporphyria (EPP) - AT SCENESSE® PASS-001: A Post-Authorisation Disease Registry Safety Study to Generate Data on the Long-Term Safety and Clinical Effectiveness of SCENESSE® (Afamelanotide 16mg) in Patients with Erythropoietic Protoporphyria (EPP) – AT	Afamelanotide 16mg	n.a.	n.a.

Currently ongoing trials are marked in green.

Table S3a: Clinical trial protocols of pharmacotherapies currently tested in erythropoietic protoporphyria

Afamelanotide

7a. Other Study ID Numbers	CUV052; NCT06388642<br>**Pharmacokinetics – Belgium, The Netherlands**	PICO
Titel	**Pharmacokinetics of Afamelanotide in EPP Patients**	
Conditions	Erythropoietic Protoporphyria (EPP)	P
Age	12 Years to 70 Years (Child, Adult, Older Adult)	P
Sexes	All	P
Enrolment	28	
Intervention/Treatment	Afamelanotide 16mg implant	I
Phase	1/2	
Inclusion Criteria	• EPP patients aged between 12 and 70 years<br>• BMI between 15 and 30 kg/m2<br>• >50 kg	P
Exclusion Criteria	• Any personal or direct family history of melanoma<br>• Any significant history of allergy and/or sensitivity to any of the contents of study drug product or lignocaine or other local anaesthetics if used<br>• Any significant illness during the four weeks before the study screening period<br>• Any evidence of hepatic or renal impairment	Safety
Primary Purpose	Other	
Allocation	N/A	
Interventional Model	Single Group Assignment	C
Arms and Interventions	Experimental: Afamelanotide 16 mg implant	I
Masking	None (Open Label)	C
Primary Outcome Measures	Cmax (maximum Plasma Concentration): Blood samples will be collected for analysis of afamelanotide plasma concentrations and understand what the maximum concentration in plasma is. Baseline to Day 7	O
	AUC(0-t) (area under the curve from administration to last observed concentration at time t). Blood samples will be collected for analysis of afamelanotide plasma concentrations. Baseline to Day 7	O
Secondary Outcome Measures	AUC(0-∞) (area under the curve extrapolated to infinite time). Baseline to Day 7	O

Study Sites	Belgium, The Netherlands	

7b. Other Study ID Numbers	CUV052; NCT06388642<br>**Pharmacokinetics – Switzerland**	PICO
Titel	**A study to evaluate the pharmacokinetics of Afamelanotide in patients with erythropoietic protoporphyria (EPP)**	
Conditions	Erythropoietic Protoporphyria (EPP)	P
Age	12 Years to 70 Years	P
Sexes		
Enrolment		
Intervention/Treatment	Afamelanotide 16mg implant	I
Phase		
Inclusion Criteria	• Fourteen adult EPP patients aged between 18 and 70 years (inclusive) and fourteen EPP adolescents aged between 12 and 17 years (inclusive).<br>• • >50 kg<br>• Participants are able and willing to comply with the protocol requirements, including refraining from the use of tanning products and excessive exposure to ultraviolet (UV) light from the start of the study until day 90. (BASEC)	P
Exclusion Criteria	• Significant history of allergies and/or sensitivity to any of the components of the investigational medicinal product.<br>• Administration of Afamelanotide in the last 60 days<br>• Donation of 400 ml or more of blood or significant blood loss in the eight weeks prior to screening. (BASEC)	
Primary Purpose	Summary description of the study<br><br>The aim of the CUV052 study is to determine the pharmacokinetics of Afamelanotide in patients with EPP (adolescent and adult patients) and to test the safety and tolerability of Afamelanotide in both adult and adolescent EPP patients. Afamelanotide is a synthetically produced chemical that is very similar to the natural human hormone Alpha-Melanocyte-Stimulating Hormone (α-MSH). Afamelanotide acts similarly to the natural hormone that increases skin melanization. In EPP patients, Afamelanotide activates the production of eumelanin in the melanocytes without prior cell damage in response to UV radiation. This increased production of eumelanin provides EPP patients with light	O

	protection, aiming to reduce the number and severity of phototoxic reactions they experience due to this disease while improving their quality of life. SCENESSE® (Afamelanotide 16mg implant) is approved for the treatment of EPP patients aged 18 years and older in the EU, Australia, and the USA. Each patient receives an Afamelanotide implant and is monitored over a period of 90 days. Throughout the study, patients will also undergo safety assessments, including blood and urine analyses, vital signs, and checks of concomitant medication and adverse events.	
Allocation		
Interventional Model		
Arms and Interventions	SCENESSE® (Afamelanotide 16 mg implant)	I
Masking		
Primary Outcome Measures		
Secondary Outcome Measures		
Study Sites	Zurich	

6. Study ID Number	NCT04578496<br>**OLE**	PICO
Other Study ID Numbers	CUV037	
Titel	**A Safety Extension Study in Patients With Erythropoietic Protoporphyria (EPP)**	
Conditions	Erythropoietic Protoporphyria (EPP)	
Age	18 Years to 75 Years (Adult, Older Adult)	P
Sexes	All	P
Enrolment	16	
Intervention/Treatment	Afamelanotide	I
Phase	3	
Inclusion Criteria	• male or female subjects with a positive diagnosis of EPP who successfully completed the CUV017 or CUV029 studies;<br>• aged 18-75 years (inclusive);<br>• provide written informed patient consent prior to the performance of any study-specific procedure.	P
Exclusion Criteria	• any serious adverse event considered to be related to afamelanotide or the polymer contained in the implant;	Safety

	• any allergy to lignocaine or other local anaesthetic to be used during the administration of the study medication;<br>• EPP patients with significant hepatic involvement;<br>• personal history of melanoma or dysplastic nevus syndrome;<br>• current Bowen's disease, basal cell carcinoma, squamous cell carcinoma, or other malignant or premalignant skin lesions;<br>• any evidence of clinically significant organ dysfunction or any clinically significant deviation from normal in the clinical or laboratory determinations;<br>• acute history of drug or alcohol abuse (in the last 12 months);<br>• female who is pregnant (confirmed by positive serum β-Human Chorionic Gonadotropin (HCG) pregnancy test prior to baseline) or lactating;<br>• females of child-bearing potential (pre-menopausal, not surgically sterile) not using adequate contraceptive measures (i.e. oral contraceptives, diaphragm plus spermicide, intrauterine device);<br>• sexually active men with partners of child-bearing potential not using barrier contraception during the trial and for a period of three months thereafter;<br>• participation in a clinical trial for an investigational agent within 30 days prior to the screening visit;<br>• prior and concomitant therapy with medications which may interfere with the objectives of the study.	
Primary Purpose	Treatment	
Allocation	N/A	
Interventional Model	Single Group Assignment	C
Arms and Interventions	Afamelanotide	I
Masking	None (Open Label)	C
Primary Outcome Measures	Change in Quality of Life Measured Using the EPP-quality of Life Questionnaire (EPP-QoL).: The EPP-QoL is presented as 15 questions aiming at measuring how the condition has affected the patient over the last 2 months. Each question is answered on a categorical scale. The maximum score is calculated by summing the score of each question resulting in a maximum of 100 and a minimum of 0. The higher the score, the better the quality of life. Baseline, Day 60, Day 120, Day 180 or early termination (ET) (if applicable)	O/ PROM /QoL
	Change in Quality of Life Measured Using the Dermatology Life Quality Index Questionnaire (DLQI). The DLQI questionnaire consists of 10 questions, each answered	O/ PROM

	on a categorical scale. The DLQI is calculated by summing the score of each question resulting in a maximum of 30 and a minimum of 0. The higher the score, the more quality of life is impaired. Baseline, Day 60, Day 120, Day 180 or early termination (ET) (if applicable)	/QoL
Study Sites	Location not provided	

5. Study ID Number	NCT04053270<br>**RCT**	PICO
Other Study ID Numbers	CUV017	
Titel	**Multicentre Phase III Erythropoietic Protoporphyria Study**	
Conditions	Erythropoietic Protoporphyria (EPP).	P
Age	18 Years to 70 Years (Adult, Older Adult)	P
Sexes	All	
Enrolment	100	
Intervention/Treatment	Afamelanotide	I
Phase	3	
Inclusion Criteria	• Male or female patients with a diagnosis of EPP (confirmed by elevated free protoporphyrin in peripheral erythrocytes) of sufficient severity that they have requested treatment to alleviate their symptoms.<br>• Aged 18-70 years.<br>• Written informed consent prior to the performance of any study-specific procedure.	P
Exclusion Criteria	• Any allergy to afamelanotide or the polymer contained in the implant or to lignocaine or other local anaesthetic used during the administration of study medication.<br>• EPP patients with significant hepatic involvement.<br>• Personal history of melanoma or dysplastic nevus syndrome.<br>• Current Bowen's disease, basal cell carcinoma, squamous cell carcinoma, or other malignant or premalignant skin lesions.<br>• Any other photodermatosis such as PLE, DLE or solar urticaria.<br>• Diagnosed with HIV/AIDS or hepatitis.<br>• Any evidence of clinically significant organ dysfunction or any clinically significant deviation from normal in the clinical or laboratory determinations.<br>• Acute history of drug or alcohol abuse (in the last 12 months).	Safety

	• History of disorders of the gastrointestinal, hepatic, renal, cardiovascular, respiratory, endocrine (including diabetes, Cushing's syndrome, Addison's disease, Peutz-Jeagher syndrome), neurological (including seizures), haematological (especially anaemia of less than 10 g/100 mL) or systemic disease judged to be clinically significant by the Investigator.<br>• Major medical or psychiatric illness<br>• Patient assessed as not suitable for the study in the opinion of the investigator (e.g. noncompliance history allergic to local anaesthetics, faints when given injections or giving blood).<br>• Female who was pregnant (confirmed by positive serum β-HCG pregnancy test prior to baseline) or lactating.<br>• Females of child-bearing potential (pre-menopausal, not surgically sterile) not using adequate contraceptive measures (i.e. oral contraceptives, diaphragm plus spermicide, intrauterine device).<br>• Participation in a clinical trial of an investigational agent within 30 days prior to the screening visit.<br>• Use of regular medications as specified in protocol Section 5.4 Prior and Concomitant Therapy.<br>• Any factors that may affect skin reflectance measurements.	
Primary Purpose	Treatment	
Allocation	Randomized	C
Interventional Model	Crossover Assignment	C
Arms and Interventions	Group A was administered active implants on Days 0, 120, 240 and placebo implants on Days 60, 180, 300.<br>Group B was administered placebo implants on Days 0, 120, 240 and active implants on Days 60, 180, 300.	C
Masking	Quadruple (ParticipantCare ProviderInvestigatorOutcomes Assessor)	
Primary Outcome Measures	Cumulative Number of Days of Phototoxic Reactions (Study Efficacy Population).<br><br>The cumulative number of days where a phototoxic reaction occurred was recorded in the patient diary. The reported data represent a cumulative total for days of phototoxic reactions.	O

	The participant scored the level of pain using an 11-point Likert pain scale, with minimum of 0 and maximum of 10. The 11-point Likert Pain scale with a value of 0 represents no pain and 10 represents worst imaginable pain.<br><br>The primary analysis population for efficacy was revised, to analyze only participants who reported cumulative total Likert pain scores of at least 26 during the study (0-360 days). This population, which was comprised of 60 patients, is identified as the Efficacy population. Participants with less than 26 Likert pain scores were not included in the analysis.<br><br>0-360 days or Early Termination	
	The Mean Number of Phototoxic Reactions (Study Efficacy Population).<br><br>The mean number of phototoxic reactions that occurred whilst patients were on active compared with placebo implants.<br><br>The days on which the participant experienced pain as a result of phototoxic reactions (caused by exposure to natural light) was recorded in a study diary. On each day such a reaction occurred, the participant scored the level of pain using an 11-point Likert pain scale, with minimum of 0 and maximum of 10. The 11-point Likert pain scale with a value of 0 represents no pain and 10 represents worst imaginable pain.<br><br>The primary analysis population for efficacy was revised, to analyze only participants who reported a cumulative total Likert pain score of at least 26 during the study (0-360 days). This population, which was comprised of 60 patients, is identified as the Efficacy population.<br><br>Participants with less than 26 Likert pain scores were not included in the analysis.<br><br>0-360 days or Early Termination	O
Secondary Outcome Measures	Cumulative Number of Days With Sunlight Exposure (Study Efficacy Population)<br><br>The number of days with sunlight exposure was recorded in the patient diary. The sunlight exposures were divided into the following categories: none, < 1 hour, 1 to 3 hours, 3 to 6 hours and > 6 hours per day.	O

	0-360 days or Early Termination	
V2	Skin Melanin Density (Study Completers Population)<br><br>Changes in melanin density (MD) (measured by spectrophotometry) at each visit by group.<br><br>Participants had their skin pigmentation measured by a non-invasive quantitative skin chromaticity (reflectance) reading. Reflectance by the skin of light measured at the wavelengths of 400 nm and 420 nm was recorded using a Minolta cm-2500d spectrophotometer at the following skin sites: forehead, left cheek, right inside upper arm, left medial forearm, right side of abdomen (avoiding implant insertion site), left side of sacral region/buttock.<br><br>Melanin density was determined for each skin site using the method of Dwyer et al 1998.<br><br>Day0, Day14, Day30, Day60, Day74, Day90, Day120, Day150, Day180, Day210, Day240, Day270, Day300, Day330, Day360 or Early Termination	
	Change in Quality of Life Using SF36 Questionnaire (Physical Component Score) for Study Completers Population.<br><br>The Summary of SF36 change from Baseline of Physical Component Score (PCS) to the scores during treatment on Days 60, 120, 180, 240, 300 and 360 using the SF36 questionnaire.<br><br>The SF-36 (The Short Form 36 Health Survey) consists of eight scaled scores, which are the weighted sums of the questions in each section. Each scale is directly transformed into a 0-100 scale on the assumption that each question carries equal weight.<br><br>The higher scores represent better health-related quality-of-life.<br><br>Day0, Day60, Day120, Day180, Day240, Day300, Day360 or Early Termination	O/ PROM /QoL

	Change in Quality of Life Using SF36 Questionnaire (Mental Component Score) for Study Completers Population.<br><br>The Summary of SF36 change from Baseline of Mental Component Score (MCS) to the scores during treatment on Days 60, 120, 180, 240, 300 and 360 using the SF36 questionnaire.<br><br>The SF-36 (The Short Form 36 Health Survey) consists of eight scaled scores, which are the weighted sums of the questions in each section. Each scale is directly transformed into a 0-100 scale on the assumption that each question carries equal weight.<br><br>The higher scores represent better health-related quality-of-life.<br><br>Day0, Day60, Day120, Day180, Day240, Day300, Day360 or Early Termination	O/ PROM /QoL
Study Sites	Location not provided	

**4. Study ID Number**	**NCT01605136 – Pivotal study -- main trial included in the analysis -- RCT**	**PICO**
Other Study ID Numbers	CUV039	
Titel	**Phase III Confirmatory Study in Erythropoietic Protoporphyria**	
Conditions	Erythropoietic Protoporphyria (EPP)	P
Age	18 Years and older (Adult, Older Adult)	P
Sexes	All	P
Enrolment	93	
Intervention/Treatment	Afamelanotide	I
Phase	3	
Inclusion Criteria	• Male or female subjects with characteristic symptoms of EPP phototoxicity and a biochemically-confirmed diagnosis of EPP.<br>• Aged 18 years old and above (inclusive).<br>• Able to understand and sign the written Informed Consent Form.<br>• Willing to take precautions to prevent pregnancy until completion of the study (Day 180).	P

Exclusion Criteria	• Any allergy to afamelanotide or the polymer contained in the implant or to lidocaine or other local anesthetic to be used during the administration of the study medication<br>• EPP patients with significant hepatic involvement<br>• Personal history of melanoma or dysplastic nevus syndrome.<br>• Current Bowen's disease, basal cell carcinoma, squamous cell carcinoma, or other malignant or premalignant skin lesions.<br>• Any other photodermatosis such as polymorphic light eruption, actinic prurigo, discoid lupus erythematosus, chronic actinic dermatitis or solar urticaria.<br>• Any evidence of clinically significant organ dysfunction or any clinically significant deviation from normal in the clinical or laboratory determinations.<br>• Acute history of drug or alcohol abuse (in the last 6 months).<br>• Patient assessed as not suitable for the study in the opinion of the Investigator (e.g. noncompliance history, allergic to local anesthetics, faints when given injections or giving blood).<br>• Participation in a clinical trial for an investigational agent within 30 days prior to the screening visit.<br>• Prior and concomitant therapy with medications which may interfere with the objectives of the study, including drugs that cause photosensitivity or skin pigmentation.<br>• Female who is pregnant (confirmed by positive serum β-HCG pregnancy test prior to baseline) or lactating.<br>• Females of child-bearing potential (pre-menopausal, not surgically sterile) not using adequate contraceptive measures (i.e. oral contraceptives, diaphragm plus spermicide, intrauterine device).	Safety
Primary Purpose	Treatment	
Allocation	Randomized	
Interventional Model	Parallel Assignment	C
Arms and Interventions	Afamelanotide, Placebo	I; C
Masking	Quadruple (ParticipantCare ProviderInvestigatorOutcomes Assessor)	
Primary Outcome Measures	Duration of Direct Sunlight Exposure Between 10:00 and 18:00 Hours on Days When no Pain Was Experienced (Pain Score of 0).	O

	The amount of direct sunlight exposure between 10:00 and 18:00 hours on days when no pain was experienced (e.g.11-point Likert pain score of 0). Time was recorded in a patient diary using 15 minute time blocks.<br><br>The pain score is measured by the 11-point Likert Pain scale with minimum of 0 and maximum of 10. Likert Pain scale of 0 represents no pain and 10 represents worst imaginable pain.<br><br>Daily for 6 months	
Secondary Outcome Measures	Combined Sun Exposure and Phototoxic Pain<br><br>Time in direct sunlight exposure between 10:00 and 18:00 hours on days when no or mild pain was experienced (Likert scores of 0 to 3).<br><br>The pain score is measured by the 11-point Likert Pain scale with minimum of 0 and maximum of 10.<br><br>Likert Pain scale of 0 represents no pain and 10 represents worst imaginable pain.<br><br>Daily for 6 months	O
Sun Exposure	Duration of direct sunlight exposure between 10:00 and 18:00 hours during the study.	O
Quality of Life Score	The Quality of life of participant is measured using DLQI and EPP QoL.<br><br>The Dermatology Life Quality Index (DLQI) is a simple practical measure for routine clinical use. The DLQI ranges from 0 (no impact on life) to 30 (significant impact on life). The Erythropoietic protoporphyria quality of life measure (EPP-QoL) scores range from 0 (worst imaginable QoL) to 100 (best possible QoL).<br><br>Day 60, Day 120, and Day 180 or early termination.	O/ PROM, QoL
Photoprovocation	Photoprovocation: A subset of subjects was photoprovoked on the dorsal surface of the hand (predilection place) and lower back and the minimum symptom dose (MSD) determined on Days 0, 30, 60, 90 and 120.	O

	The amount of radiation required to provoke the first clinical symptom was recorded.<br><br>Day 0, Day 30, Day 60, Day 90 and Day 120.	
Maximum Severity of Phototoxic Reaction Experienced by Participants	Maximum Severity of Phototoxic Reaction Experienced by Participants. The phototoxicity - phototoxic pain secondary endpoint has been divided into two secondary outcome measures.<br><br>The days on which the participant experienced pain as a result of phototoxic reactions (caused by exposure to natural light) was recorded in a study diary. On each day such a reaction occurred, the participant scored the level of pain using an 11-point Likert pain scale, with minimum of 0 and maximum of 10. The 11-point Likert pain scale with a value of 0 represents no pain and 10 represents worst imaginable pain.<br><br>The maximum severity of a phototoxic reaction was determined by the highest daily 11-point Likert scale score that occurred during that phototoxic reaction.<br><br>Daily for 6 months	O
Total Number Phototoxic Reactions Experienced by Participants	Total Number Phototoxic Reactions Experienced by Participants.<br><br>The phototoxicity - phototoxic pain secondary endpoint has been divided into two secondary outcome measures.<br><br>The number of episodes was the endpoint. The days on which the participant experienced pain as a result of phototoxic reactions (caused by exposure to natural light) was recorded in a study diary. On each day such a reaction occurred, the participant scored the level of pain using an 11-point Likert pain scale, with minimum of 0 and maximum of 10. The 11-point Likert Pain scale with a value of 0 represents no pain and 10 represents worst imaginable pain.<br><br>The number of phototoxic reactions was determined by counting the number of episodes on which participants report a 11-point Likert scale score of 4 or more for one or more consecutive days.<br><br>Daily for 6 months	O
Study Sites	USA	

3. Study ID Number	NCT01097044<br>**RCT**	PICO
Other Study ID Numbers	CUV030	
Titel	**Phase II Confirmatory Study in Erythropoietic Protoporphyria (EPP)**	
Conditions	Erythropoietic Protoporphyria (EPP)	
Age	18 Years and older (Adult, Older Adult)	P
Sexes	All	P
Enrolment	77	
Intervention/Treatment	Afamelanotide, Placebo	I; C
Phase	2	
Inclusion Criteria	• Male or female subjects with characteristic photosensitivity of EPP symptoms and positive diagnosis of EPP confirmed by laboratory result of elevated total protoporphyrin IX.<br>• Aged 18 years old and above (inclusive).<br>• Able to understand and sign the written Informed Consent Form.<br>• Willing to take precautions to prevent pregnancy until completion of the study (Day 180).	P
Exclusion Criteria	• Any allergy to afamelanotide or the polymer contained in the implant or to lidocaine or other local anesthetic to be used during the administration of the study medication<br>• EPP patients with significant hepatic involvement<br>• Personal history of melanoma or dysplastic nevus syndrome.<br>• Current Bowen's disease, basal cell carcinoma, squamous cell carcinoma, or other malignant or premalignant skin lesions.<br>• Any other photodermatosis such as PLE, DLE or solar urticaria.<br>• Any evidence of clinically significant organ dysfunction or any clinically significant deviation from normal in the clinical or laboratory determinations.<br>• Acute history of drug or alcohol abuse (in the last 6 months).<br>• Patient assessed as not suitable for the study in the opinion of the Investigator (e.g. noncompliance history, allergic to local anesthetics, faints when given injections or giving blood).<br>• Participation in a clinical trial for an investigational agent within 30 days prior to the screening visit.	Safety

	• Prior and concomitant therapy with medications which may interfere with the objectives of the study, including drugs that cause photosensitivity or skin pigmentation within 60 days prior to the screening visit.<br>• Female who is pregnant (confirmed by positive serum β-HCG pregnancy test prior to baseline) or lactating.<br>• Females of child-bearing potential (pre-menopausal, not surgically sterile) not using adequate contraceptive measures (i.e. oral contraceptives, diaphragm plus spermicide, intrauterine device).	
Primary Purpose	Treatment	
Allocation	Randomized	
Interventional Model	Parallel Assignment	
Arms and Interventions	Afamelanotide, Placebo	I; C
Masking	Quadruple (ParticipantCare ProviderInvestigatorOutcomes Assessor)	
Primary Outcome Measures	Time in Direct Sunlight Between 10:00-15:00 on Pain-free Days.<br><br>The amount of direct sunlight exposure between 10:00 and 15:00 hours on days when no pain was experienced (e.g. 11-point Likert pain score of 0). Time was recorded in a patient dairy using 15 minute time blocks.<br><br>The pain score is measured by the 11-point Likert Pain scale with minimum of 0 and maximum of 10.<br><br>Likert Pain scale of 0 represents no pain and 10 represents worst imaginable pain.<br><br>Daily for 6 months	O
Secondary Outcome Measures	Maximum Severity of Phototoxic Reaction Experienced by Participants.<br><br>The days on which the participant experienced pain as a result of phototoxic reactions (caused by exposure to natural light) was recorded in a study diary. On each day such a reaction occurred, the participant scored the level of pain using an 11-point Likert pain scale, with minimum of 0 and maximum of 10. The 11-point Likert pain scale with a value of 0 represents no pain and 10 represents worst imaginable pain.	O

	The maximum severity of a phototoxic reaction was determined by the highest daily 11-point Likert scale score that occurred during that phototoxic reaction.<br><br>Daily for 6 months	
	Quality of Life Measured by Participant Completed Questionnaire.<br><br>Erythropoietic Protoporphyria Quality of Life Measure (EPP-QoL) is used to measure the quality of life of participants.<br><br>The total EPP-QoL score ranges from 0 to 100, with a score of 0 as the worst quality of life and score of 100 as the best quality of life.<br><br>Day 0, Day 60, Day 120, Day 180	O/ PROM<br><br>/QoL
	Change of Total Protoporphyrin IX Level in Participants. This was an exploratory assessment only to analyze whether afamelanotide-induced change in sun exposure would result in a reduction of protoporphyrin IX.<br><br>The changes of the Total Protoporphyrin IX Level (μg/dL) from Screening Visit (ITT Population) were measured between the two groups.<br><br>The Protoporphyrin IX level is a laboratory parameter that is measured in specialist labs.<br><br>Baseline, Day 60, Day 120, Day 180	O
	Number of Participants With Phototoxic Reactions With Likert Severity Scores ≥ 4 and ≥ 7.<br><br>The number of participants who experienced phototoxic reactions with Likert severity scores ≥ 4 and severity scores ≥7 were recorded.<br><br>A derived endpoint was used. The number of participants who reported at least one phototoxic reaction with a Likert severity score of ≥ 4 was recorded. For severity scores ≥ 7, the number of patients who reported at least one phototoxic reaction with a Likert severity score of ≥ 7 was recorded.	O

	The 11-point Likert pain scale ranges from minimum of 0 to maximum of 10. The 11-point Likert pain scale with a value of 0 represents no pain and 10 represents worst imaginable pain.<br><br>Daily for 6 months	
	Number of Phototoxic Reactions Experienced by Participants.<br><br>The days on which the participant experienced pain as a result of phototoxic reactions (caused by exposure to natural light) was recorded in a study diary. On each day such a reaction occurred, the participant scored the level of pain using an 11-point Likert pain scale, with minimum of 0 and maximum of 10. The 11-point Likert pain scale with a value of 0 represents no pain and 10 represents worst imaginable pain.<br><br>The number of phototoxic reactions was determined by counting the number of episodes on which participants report a 11-point Likert scale score of 4 or more for one or more consecutive days.<br><br>Daily for 6 months	O
	Number of Phototoxic Reactions Experienced by Participants.<br><br>The days on which the participant experienced pain as a result of phototoxic reactions (caused by exposure to natural light) was recorded in a study diary. On each day such a reaction occurred, the participant scored the level of pain using an 11-point Likert pain scale, with minimum of 0 and maximum of 10. The 11-point Likert pain scale with a value of 0 represents no pain and 10 represents worst imaginable pain.<br><br>The number of phototoxic reactions was determined by counting the number of episodes on which participants report a 11-point Likert scale score of 4 or more for one or more consecutive days.<br><br>Daily for 6 months	O
Total Severity of Phototoxic Reactions Experienced by Participants Over the Entire Study	The days on which the participant experienced pain as a result of phototoxic reactions (caused by exposure to natural light) was recorded in a study diary.<br><br>On each day such a reaction occurred, the participant scored the level of pain using an 11-point Likert pain scale, with minimum of 0 and maximum of 10. The 11-point Likert	O

	Pain scale with a value of 0 represents no pain and 10 represents worst imaginable pain.<br><br>The total severity of phototoxic reactions was determined by the sum of daily 11-point Likert scale scores that occurred during phototoxic reactions. The overall sum of the severity per participant over the entire study was analyzed. The theoretical minimum score is 0 and the maximum possible score is 1800.<br><br>Daily for 6 months.	
Study Sites	USA	

2. Study ID Number	NCT00979745<br>**RCT**	PICO
Other Study ID Numbers	CUV029	
Titel	**Phase III Confirmatory Study in Erythropoietic Protoporphyria (EPP)**	
Conditions	Erythropoietic Protoporphyria (EPP)	P
Age	18 Years to 70 Years (Adult, Older Adult)	P
Sexes	All	P
Enrolment	74	
Intervention/Treatment	Afamelanotide	
Phase	3	
Inclusion Criteria	• Male or female subjects with a diagnosis of EPP (confirmed by elevated free protoporphyrin in peripheral erythrocytes) of sufficient severity that they have requested treatment to alleviate their symptoms.<br>• Aged 18 - 70 years (inclusive)<br>• Written informed consent prior to the performance of any study-specific procedures.	P<br><br>Safety
Exclusion Criteria	• Any allergy to afamelanotide or the polymer contained in the implant or to lignocaine or other local anaesthetic to be used during the administration of study medication.<br>• EPP patients with significant hepatic involvement.<br>• Personal history of melanoma or dysplastic nevus syndrome.	

	• Current Bowen's disease, basal cell carcinoma, squamous cell carcinoma, or other malignant or premalignant skin lesions.<br>• Any other photodermatosis such as PLE, DLE or solar urticaria.<br>• Any evidence of clinically significant organ dysfunction or any clinically significant deviation from normal in the clinical or laboratory determinations.<br>• Acute history of drug or alcohol abuse (in the last 12 months).<br>• Patient assessed as not suitable for the study in the opinion of the Investigator (e.g. noncompliance history, allergic to local anaesthetics, faints when given injections or giving blood).<br>• Female who is pregnant (confirmed by positive serum β-HCG pregnancy test prior to baseline) or lactating.<br>• Females of child-bearing potential (pre-menopausal, not surgically sterile) not using adequate contraceptive measures (i.e. oral contraceptives, diaphragm plus spermicide, intrauterine device).<br>• Sexually active men with partners of child bearing potential not using barrier contraception during the trial and for a period of three months thereafter.<br>• Participation in a clinical trial of an investigational agent within 30 days prior to the screening visit.<br>• Prior and concomitant therapy with medications which may interfere with the objectives of the study, including drugs that cause photosensitivity or skin pigmentation.	
Primary Purpose	Prevention	
Allocation	Randomized	
Interventional Model	Parallel Assignment	
Arms and Interventions	Afamelanotide, Placebo	I; C
Masking	Quadruple (ParticipantCare ProviderInvestigatorOutcomes Assessor)	
Primary Outcome Measures	The Duration of Direct Sunlight Exposure Between 10:00 and 15:00 Hours on Days When Patients Did Not Report Phototoxicity-related Pain (Likert Pain Scale Score of 0). From baseline to Day 270	O
Secondary Outcome Measures	Number of Phototoxic Reactions. 9 months	O
	Quality of Life Measured by Patient Completed Questionnaire. 9 months	
	Free Protoporphyrin IX Level. 9 months	O
	Treatment Emergent Adverse Events. 9 months	Safety
Study Sites	Finland, France, Germany, Ireland, Netherlands, UK	

1. Study ID Number	ACTRN12606000535572<br>**OL**	PICO
Other Study ID Numbers	CUV010<br>HumRes6296	
Titel	**A Multicentre, Phase II, Open Label Study to Evaluate the Safety of CUV1647 and to Evaluate the Effect of Subcutaneous Implants of CUV1647 on the Time to Artificially Provoked Symptoms in Patients with Erythropoietic Protoporphyria (EPP)**	
Conditions	Erythropoietic Protoporphyria (EPP)	P
Age	18 to 70	P
Sexes	Both males and females	P
Enrolment		
Intervention/Treatment	Afamelanotide, 20 mg	I
Phase	2	
Inclusion Criteria	Patients with EPP (confirmed by elevated free protoporphyrin in peripheral erythrocytes and/or ferrochelatase mutation)- Fitzpatrick skin types I to IV.<br><br>Minimum age: 18 Years<br><br>Maximum age: 70 Years<br><br>Gender: Both males and females	P
Exclusion Criteria	Any other photodermatosis such as polymorphic light eruption (PLE), discoid lupus erythematosus (DLE) or solar urticaria<br><br>- females who are pregnant, lactating or of child bearing age not using adequate methods of contraception<br><br>- Any evidence of clinically significant organ dysfunction or any clinically significant deviation from normal in the clinical or laboratory determinations<br><br>- Acute history of drug or alcohol abuse (in the last 12 months)	P

	- History of disorders of the gastrointestinal, hepatic, renal, cardiovascular, respiratory, endocrine, neurological, haematological or systemic disease judged to be clinically significant by the Investigator.	
Primary Purpose	The study is intended to determine if CUV1647 can increase the tolerance of patients with EPP to sunlight and improve their quality of life.	O
Allocation		
Interventional Model		
Arms and Interventions	OL	C
Masking		
Primary Outcome Measures		
Secondary Outcome Measures		
Study Sites	Zurich, Switzerland	

**PASS:**<br><br>**Study ID Number**	**CUV-PASS-001**	**PICO**
Other Study ID Numbers	SCENESSE ® PASS v8: 7 March 2016	
Titel	A Post-Authorisation Disease Registry Safety Study to Generate Data on the Long-Term Safety and Clinical Effectiveness of SCENESSE® (Afamelanotide 16mg) in Patients with Erythropoietic Protoporphyria (EPP).	I
Condition	Erythropoietic Protoporphyria (EPP).	P
Age	Aged 18 years or more	P
Sexes		
Enrolment	EPP is an ultra-orphan indication (estimated to affect less than 1 in 50,000 individuals) therefore patient numbers in the study will be limited. It is anticipated that in the first two years the study will enrol 200 patients from an estimated	P

	eligible population of 660 patients with EPP. It is likely that at most 10% of these will not receive SCENESSE® treatment. The study will run indefinitely.	
Intervention/Treatment	This is a non-interventional post-authorisation registry study.	
Phase	Phase IV	
Inclusion criteria	To be eligible for treatment, patients must meet the following criteria:<br>- Patients with erythropoietic protoporphyria (EPP)<br>- Aged 18 years or more<br>- Patient where treatment is not contraindicated in accordance with the approved<br>SmPC.	P
Exclusion criteria	Non-eligibility Criteria for Treatment<br>If the following SCENESSE® contraindications apply, the patient is not eligible for treatment,<br>but can still be enrolled in the untreated ‘comparator’ group:<br>- Allergy or hypersensitivity to the active substance or to any of the excipients listed in<br>section 6.1 of SmPC<br>- Presence or history of severe hepatic disease<br>- Hepatic impairment<br>- Renal impairment<br>- Children and adolescents (0-17 years)	P

	Women of childbearing potential should use effective contraception during treatment with SCENESSE® and for a period of three months thereafter. SCENESSE® should not be used during pregnancy.<br><br>It is unknown whether afamelanotide or any of its metabolites are excreted in breast milk, so SCENESSE® should be avoided during breastfeeding.<br><br>Additionally, a careful clinical decision must be made whether to treat patients with any clinically significant disorders of the gastrointestinal, cardiovascular, respiratory, endocrine (including diabetes, Cushing's disease, Addison's disease, Peutz-Jeghers syndrome), neurological (including seizures) and haematological (especially anaemia) systems. If such patients are treated they must be monitored closely after each administration, including vital signs, haematology and biochemistry.<br><br>SCENESSE® should not be used in patients over 70 years of age. If such patients are treated, they must be monitored after each administration, including vital signs, routine haematology and biochemistry.<br><br>Full a matrix of all study procedures, please refer to Appendix G.	
Primary objectives	Gather long-term safety data on SCENESSE® with respect to:<br><br>- Characterisation of the long-term safety profile with regard to all adverse events<br><br>- Changes in cutaneous efflorescence and pigmentary expressions (type, incidence and severity, and determine if there is a change of incidence or the emergence of new adverse events following repeated administration, including occurrence of skin cancer or precursors)<br><br>- Application site reactions (type, incidence and severity, and determine if there is a change of incidence or the emergence of new adverse events following repeated administration)	O

	- Allergy and hypersensitivity (type, incidence and severity, and determine if there is a change of incidence or the emergence of new adverse events following repeated administration)<br><br>- Administration errors (identify if any errors occur and if there are any associated adverse events).<br><br>Evaluate compliance with the risk minimization measures:<br><br>- Cutaneous efflorescence and pigmentary expressions - compliance with 6 monthly full body examinations<br><br>- Application site reactions – compliance with the provision of educational material, and the training and accreditation of all physicians who will administer SCENESSE®<br><br>- Off-label use - undertake routine assessments of database to determine the number of cases of use in children or adults without EPP<br><br>- Use in pregnancy or lactation – undertake routine assessments of database to determine the number of cases of use during pregnancy or lactation<br><br>- Administration error - compliance with the provision of educational material, and the training and accreditation of all physicians who will administer SCENESSE	
Secondary objective	Evaluate adherence to the controlled distribution program:<br><br>- This will be done through drug accountability records with quantities of SCENESSE® shipped to treatment centres compared with records of administered SCENESSE® and institutional pharmacy stocks (a “mass balance” will be determined)<br><br>- Given the limited number of patients likely to be recruited per treatment centre, distribution to institutional pharmacies will be done using small shipments. Records of SCENESSE® shipped will be available for individual batches and the	O

	batch number of each SCENESSE® administered will be recorded on the patient's CRF. Regular reports of shipments and SCENESSE® administered by patient number and by batch will be generated from the database. These data will be used to produce full accountability reports.<br><br>Generate data to contribute to knowledge about clinical benefits and to add data on potential clinical effectiveness of SCENESSE®<br><br>- Longitudinal assessment of activities able to be undertaken prior to treatment and those possible following commencement of treatment (using the Daily Activity Inventory)<br><br>- Longitudinal assessment of those remaining on treatment together with logs detailing reasons for discontinuing (and those recommencing) treatment as well as those declining treatment will be done using descriptive statistics. Reasons for declining treatment, discontinuing treatment and recommencing treatment will be categorised and frequency tables generated<br><br>- Quality of Life assessment using EPP-QoL questionnaire<br><br>- Sun protection measures employed and number and severity of phototoxic reactions experienced	PROM<br><br>PROM
Variables	Safety variables:<br><br>Characterisation of the overall long-term safety profile with regard to all adverse events will be undertaken. Treatment-emergent adverse events will be summarized by MedDRA preferred term and body system, and these will be further summarized by intensity, seriousness and relationship to study medication. In addition to assessing the overall adverse event profile, there will be a particular emphasis on:<br><br>- Use during pregnancy and lactation<br><br>- changes in cutaneous efflorescence and pigmentary expressions	

	- application site reactions<br>- hypersensitivity and allergy<br>Clinical effectiveness variables:<br>The clinical effectiveness of treatment with SCENESSE® will be evaluated by a number of<br>tools/questionnaires which will measure/record any changes to:<br>- Longitudinal assessment of those remaining on treatment<br>- Quality of Life (EPP-QoL)<br>- Daily Activity Inventory<br>- Sun protection measures and phototoxicity (number and severity of events)<br>Risk minimisation measures:<br>Compliance with risk minimization recommendations will be measured through the Disease<br>Registry for the following:<br>- off-label use in adults and paediatric patients<br>- use in pregnancy and lactation<br>- administration errors<br>- changes in cutaneous efflorescence and pigmentary expressions<br>- application site reactions.<br>Controlled distribution measures:	

	Full drug accountability records from institutional pharmacy records and physicians' SCENESSE® administration documentation will be captured through the registry.	
Data analysis	Safety Assessment<br><br>Sample Size Calculation for Safety Data<br><br>On the basis of 200 patients treated with SCENESSE® there will be a:<br><br>• >99% chance of observing at least one adverse event of a specific type if the true incidence of that specific adverse event is common (10%)<br><br>• 87% chance of observing at least one adverse event of a specific type if the true incidence of that specific adverse event is uncommon (1%)<br><br>• 18% chance of observing at least one adverse event of a specific type if the true incidence of that specific adverse event is rare (0.1%)<br><br>Safety Assessment<br><br>All patients enrolled in the registry will be included in the safety assessment. In general longitudinal comparisons will be within Treated Groups (including separate groups that take into account patients who commence or discontinue treatment) and between groups,<br><br>Treated versus Untreated.<br><br>The number of participants with treatment-emergent adverse events will be summarized by MedDRA PT and body system. Adverse events will be further summarized by intensity, seriousness and outcome. Adverse events will also be summarized by 6 monthly time intervals from entry into the registry to assess the longitudinal effect of the treatment and EPP. There will be a particular emphasis on the following types of adverse events:	O<br><br>Safety

	Changes in cutaneous efflorescence – a dermatological examination once every six months (equating roughly to the end of treatment in Year 1 and then prior to and at the end of the treatment period in subsequent treatment years) with an emphasis on pre-existing expressions, new cutaneous efflorescence and pigmentary expressions and/or sun damaged fields will be conducted by a dermatologist. Full body photography will be undertaken at the same time. Documentation of full body skin examination will be performed as per current clinical practice. Any changes in cutaneous efflorescence and pigmentary expressions deemed to require treatment will be treated appropriately and severity, causality and outcome will be assessed by the treating physician and/or dermatologist.<br><br>Application site reactions – these will be reported following each SCENESSE®<br><br>administration. Severity, causality and outcome will be assessed by the treating physician.<br><br>The incidence of application site reactions will be determined by comparing the number of reports with the number of SCENESSE® administrations. The acceptability criterion is that such reactions occur in less than 25% of administrations.<br><br>Allergy and hypersensitivity - these will be reported following each administration. Severity, causality and outcome will be assessed by the treating physician. The incidence of reactions requiring hospitalisation will be determined. Acceptability criterion is less than 10% of reactions that require hospitalisation.<br><br>Off-label use (in children or adults without EPP) – demographic data and confirmation of EPP diagnosis will be recorded at baseline. The incidence of treated patients who do not comply with the requirements of the SmPC for age or disease status will be determined.	

	Acceptability criteria are:<br><br>- off-label use in children represents not more than 1% of all cases<br><br>- use in adults who do not have EPP is not more than 5% of all cases<br><br>Use in pregnancy or lactation – reports of use during pregnancy or lactation will be collected and followed up and the acceptability criterion is less than 5% of women treated with SCENESSE® become pregnant or breastfeed during treatment.<br><br>Administration errors – these will be recorded and the acceptability criterion is that administration errors occur in no more than 5% all prescriptions.<br><br>Assessment of Clinical Effectiveness<br><br>Continuity on treatment - together with logs detailing reasons for discontinuing (and those recommencing)<br><br>Quality of Life (EPP-QoL) – will be measured using the EPP-QoL questionnaire, provided to the patient for completion at baseline prior to treatment in Year 1 and at each subsequent visit.<br><br>Daily Activity Inventory – will be evaluated using the questionnaire provided to the patient for completion at baseline prior to treatment in Year 1 and at each subsequent visit.<br><br>In addition, patients' statements on their experience with SCENESSE® will be recorded at the end of each treatment year.<br><br>Light and Sun Protection Measures – patients will be asked to report the sun protection measures employed.<br><br>Phototoxicity – patients will be asked to report the number and severity of phototoxic reactions and episodes experienced.	

	Risk minimisation measures:<br><br>Changes in cutaneous efflorescence – compliance with 6 monthly full body examinations.<br><br>Application site reactions – compliance with the provision of educational material, and the training and accreditation of all physicians who will administer SCENESSE®. The acceptability criterion for reactions is less than 25% of administrations.<br><br>Off-label use (in children or adults without EPP) – demographic data and confirmation of EPP will be recorded at baseline. The incidence of treated patients who do not comply with the requirements of the SmPC for age or disease status will be determined. Acceptability criteria are:<br><br>- off-label use in children represents not more than 1% of all cases<br><br>- use in adults who do not have EPP is not more than 5% of all cases<br><br>Use in pregnancy or lactation – acceptability criterion is less than 5% of women treated<br><br>with SCENESSE® become pregnant or breastfeed during treatment.<br><br>Administration errors – The acceptability criterion is that administration errors occur in no more than 5% all prescriptions.	
Study sites	It is anticipated that, in the first year post-MA, distribution will be initiated in the following countries: Austria, Germany, The Netherlands, United Kingdom. Others will be entered into the study as national Competent Authority approvals are obtained.	
Source and full protocol:	Wensink D, Wagenmakers MAEM, Barman-Aksözen J, et al. Association of Afamelanotide With Improved Outcomes in Patients With Erythropoietic	

	Protoporphyria in Clinical Practice. JAMA Dermatol. 2020;156(5):570-575. doi:10.1001/jamadermatol.2020.0352	

Table S3b: Clinical trial protocols of pharmacotherapies currently tested in erythropoietic protoporphyria

Dersimelagon

4. Study ID Number	NCT06144840 – Main trial included in the analysis -- **RCT**	PICO
Other Study ID Numbers	MT-7117-A-302	
Titel	**INcreased Sun Exposure Without Pain In Research Participants With EPP or XLP (INSPIRE)**	
Conditions	Erythropoietic Protoporphyria or X-Linked Protoporphyria	P
Age	12 Years to 75 Years (Child, Adult, Older Adult)	P
Sexes	All	P
Enrolment	150	
Intervention/Treatment	Dersimelagon vs. Placebo	I; C
Phase	3	
Inclusion Criteria	1. Subjects provided written informed consent to participate. For minor subjects, both minor's assent and parental consent will be required.<br>2. Male and female subjects with a confirmed diagnosis of EPP or XLP based on medical history.<br>3. Subjects aged 12 years to 75 years, inclusive, at Screening.<br>4. Subjects are willing and able to travel to the study sites for all scheduled visits.<br>5. In the Investigator's opinion, subject can understand the nature of the study and any risks involved in participation, and willing to cooperate and comply with the protocol restrictions and requirements (including travel and receiving direct sunlight exposure as much as possible).<br>6. Female subjects who are non-lactating and have a negative urine pregnancy test at baseline visit prior to receiving the first dose of study drug.<br>7. Female subjects of childbearing potential and male subjects with partner of child-bearing potential currently using/willing to use 2 effective methods of contraception.	P
Exclusion Criteria	1. History or presence of photodermatoses other than EPP or XLP.<br>2. Subjects who are unwilling or unable to go outside in sunlight during daylight hours most days (e.g., between 1-hour post-sunrise and 1 hour pre-sunset) during the study.	P

	3. Presence or history of any hepatobiliary disease, including drug induced liver injury at screening, determined as clinically significant by the Investigator after the discussion with the Sponsor Medical Monitor.<br>4. Subjects with aspartate aminotransferase (AST), alanine aminotransferase (ALT), alkaline phosphatase (ALP) ≥ 2.0 × upper limit of normal (ULN) or total bilirubin >1.5 × ULN at Screening.<br>5. History (in the last 2 years) or presence of alcohol abuse, or abuse of illicit drugs in the opinion of the Investigator.<br>6. History of melanoma.<br>7. Presence of squamous cell carcinoma, basal cell carcinoma, or other malignant skin lesions. Any suspicious lesions or nevi will be evaluated. If the suspicious lesion or nevi cannot be resolved through biopsy or excision, the subject will be excluded from the study.<br>8. History or presence of psychiatric disease judged to be clinically significant by the Investigator and which may interfere with the study evaluation and/or safety of the subjects.<br>9. Presence of clinically significant acute or chronic renal disease or subjects with an estimated glomerular filtration rate (eGFR) <60 mL/min as calculated by the Chronic Kidney Disease Epidemiology Collaboration (CKD-EPI) creatinine equation (2021) for adults and by the Schwartz creatinine equation for adolescents (2009). Modification of Diet in Renal Disease can be used for adults per local recommendations.<br>10. Presence of any clinically significant disease or laboratory abnormality which, in the opinion of the Investigator, can interfere with the study objectives and/or safety of the subjects.<br>11. Female subjects who are pregnant, lactating, or intending to become pregnant during the study.<br>12. Treatment with any of the following medications or therapy within each period before Randomization (Visit 2);<br>o Afamelanotide within 3 months<br>o Phototherapy within 3 months<br>o Cimetidine within 4 weeks<br>o Antioxidant agents within 4 weeks, at doses which, in the opinion of the Investigator, may affect study endpoints (including but not limited to beta-carotene, cysteine, pyridoxine).<br>o Chronic treatment with any scheduled analgesic agents including, but not limited to, opioids and opioid derivatives such as morphine, hydrocodone,	Safety

	oxycodone, fentanyl, or their combination with other unscheduled analgesics or non-steroidal anti-inflammatory drug (Percocet and Vicodin-like prescription drugs) within 4 weeks.<br>○ Note: Acute use of scheduled narcotics more than 3 months prior to randomization are allowed. Non-steroidal anti-inflammatory drug, aspirin for analgesia, or prior temporary use of scheduled agents within 3 months of screening is allowed.<br>13. Dermatological treatments with any drugs or supplements which, in the opinion of the Investigator, can interfere with the objectives of the study or safety of the subjects at screening, such as, for example, tanning agents.<br>14. Subjects who participated in any previous MT-7117 clinical studies.<br>15. Previous treatment with any investigational agent such as bitopertin, within 12 weeks before Screening or 5 half-lives of the investigational product (whichever is longer).<br>16. Use of sunscreens with zinc oxide. Note: Sunscreens without zinc oxide are allowed, however their use, in frequency, quantity and body surface area should be maintained relatively stable throughout the duration of the study.<br>17. History of any hypersensitivity to the active ingredient and/or excipients (lactose monohydrate, hydroxypropylcellulose, carmellose calcium, magnesium stearate, hypromellose, titanium dioxide, talc, polyethylene glycol, iron oxide yellow, iron oxide red, and iron oxide black). (EU ONLY)<br>18. Subjects who are unable to swallow tablets or have diseases significantly affecting the gastrointestinal function such as malabsorption syndrome, resection of the stomach or small bowel, bariatric surgery procedures, symptomatic inflammatory bowel disease, or partial or complete bowel obstruction.(EU ONLY)<br>19. History of any hypersensitivity to the active ingredient and/or excipients contained in MT-7117 IMP (lactose monohydrate, hydroxypropyl cellulose, carmellose calcium, magnesium stearate, hypromellose, titanium dioxide, talc, polyethylene glycol, iron oxide yellow, iron oxide red, and iron oxide black). (UK ONLY)	
Primary Purpose	Treatment	
Allocation	Randomized	
Interventional Model	Parallel Assignment	
Arms and Interventions	Dersimelagon, Placebo	I; C
Masking	Quadruple (ParticipantCare ProviderInvestigatorOutcomes Assessor)	

Primary Outcome Measures	Change from baseline in average daily sunlight exposure time (minutes) to first prodromal symptom (burning, tingling, itching, or stinging) associated with sunlight exposure between 1 hour post-sunrise and 1 hour pre-sunset at Week 16. The comparison between MT-7117 treatment group and placebo group will be performed. Week 16	O
Secondary Outcome Measures	Patient Global Impression of Change (PGIC) at Week 16. The comparison between MT-7117 treatment group and placebo group will be performed. Week 16	O
	Total number of sunlight-induced pain events defined as prodromal symptoms (burning, tingling, itching, or stinging) with pain rating of 1-10 on the Likert scale during the 16-week double-blind treatment period. The comparison between MT-7117 treatment group and placebo group will be performed. Week 16	O
	Total number of sunlight-induced non-prodrome, phototoxic reactions during the 16-week double-blind treatment period. The comparison between MT-7117 treatment group and placebo group will be performed. Week 16	O
Study Sites	USA, Australia, Bulgaria, Czechia, France, Italy, Japan, Netherlands, Poland, Spain, UK	

3. Study ID Number	NCT05005975<br>**OLE**	PICO
Other Study ID Numbers	MT-7117-A-301	
Titel	**Extension Study to Evaluate Safety and Tolerability of Oral Dersimelagon (MT-7117) in Subjects With Erythropoietic Protoporphyria (EPP) or X-Linked Protoporphyria (XLP)**	
Conditions	Erythropoietic Protoporphyria (EPP) or X-Linked Protoporphyria (XLP)	P
Enrolment	151	
Age	12 Years to 75 Years (Child, Adult, Older Adult)	P
Sexes	All	P
Intervention/Treatment	MT-7117	I
Phase	3	
Inclusion criteria	• 1. Subjects provided written informed consent to participate. For adolescent subjects, both adolescent assent and parental consent will be provided.<br>• 2. Subjects who complete MT-7117-G01 (complete through Week 58 [Visit 12])<br>• 3. Subjects have a body weight of ≥30 kg.<br>• 4. Subjects are willing and able to travel to the study sites for all scheduled visits.	P

	• 5. In the Investigator's opinion, subject can understand the nature of the study and any risks involved in participation, and is willing to cooperate and comply with the protocol restrictions and requirements (including travel).<br>• 6. Female subjects who are non-lactating and have a negative urine pregnancy test at baseline visit prior to receiving the first dose of study drug.<br>• 7. Female subjects of childbearing potential and male subjects with partner of childbearing potential must agree to use 2 effective methods of contraception including barrier method (especially for female subjects, one method must be highly effective method)	
Exclusion Criteria:	A subject will NOT be eligible for this study if ANY of the following criteria apply:<br><br>• 1. History or presence of photodermatoses other than EPP or XLP.<br>• 2. Presence or history of any hepatobiliary disease at Screening, determined as clinically significant by the Investigator after the discussion with the Sponsor Medical Monitor.<br>• 3. Subjects with AST, ALT, ALP ≥3.0 × upper limit of normal (ULN) or total bilirubin >1.5 × ULN at Screening.<br>• 4. Subjects with or having a history (in the last 2 years) of excessive alcohol intake in the opinion of the Investigator.<br>• 5. History of melanoma.<br>• 6. Presence of melanoma and/or lesions suspicious for melanoma at Screening.<br>• 7. History of familial melanoma (defined as having 2 or more first-degree relatives, such as parents, sibling and/or child).<br>• 8. Presence of squamous cell carcinoma, basal cell carcinoma, or other malignant skin lesions. Any suspicious lesions or nevi will be evaluated. If the suspicious lesion or nevi cannot be resolved through biopsy or excision, the subject will be excluded from the study.<br>• 9. History or presence of psychiatric disease judged to be clinically significant by the Investigator and which may interfere with the study evaluation and/or safety of the subjects.<br>• 10. Presence of clinically significant acute or chronic renal disease based upon the subject's medical records including hemodialysis; an estimated glomerular filtration rate (eGFR) <60 mL/min/1.73m2 as calculated by the CKD-EPI creatinine equation (2009) for adults and by the Schwartz creatinine equation	P<br><br><br><br><br><br>Safety

	for adolescents (2009). MDRD can be used for adults per local recommendations.<br>• 11. Presence of any clinically significant disease or laboratory abnormality which, in the opinion of the Investigator, can interfere with the study objectives and/or safety of the subjects.<br>• 12. Female subjects who are pregnant, lactating, or intending to become pregnant during the study.<br>• 13. Treatment with phototherapy or afamelanotide within 3 months before baseline (Visit 2).<br>• 14. Treatment with cimetidine or antioxidant agents at doses which, in the opinion of the Investigator, may affect study endpoints (including but not limited to beta-carotene, cysteine, pyridoxine) within 4 weeks before baseline (Visit 2).<br>• 15. Chronic treatment with any scheduled analgesic agents including, but not limited to opioids and opioid derivatives such as morphine, hydrocodone, oxycodone, fentanyl, or their combination with other unscheduled analgesics or non-steroidal anti-inflammatory drug (Percocet and Vicodin-like prescription drugs) within 4 weeks before baseline (Visit 2). Acute use of scheduled narcotics greater than 3 months prior to baseline, over-the-counter medications (OTCs), such as non-steroidal anti-inflammatory drugs (NSAIDs) or aspirin for analgesia, or prior temporary use of scheduled agents within 3 months of baseline (Visit 2) are not excluded.<br>• 16. Treatment with any drugs or supplements which, in the opinion of the Investigator, can interfere with the objectives of the study or safety of the subjects.<br>• 17. Previous treatment with any investigational agent other than dersimelagon within 12 weeks before Screening OR 5 half-lives of the investigational product (whichever is longer).	
Primary Purpose	Treatment	
Allocation	N/A	
Interventional Model	Single Group Assignment	
Arms and interventions	Dersimelagon 200mg	I
Masking	None (Open Label)	C
Primary Outcome Measures	Number of patients with Treatment emergent adverse events (TEAEs) (including serious adverse events [SAEs] and adverse events of special interest [AESIs]).	O/ Safety
	Number of patients with abnormal Physical examination data	

	Number of patients with Nevi appearance	O/ Safety
Secondary Outcome Measures	n.a.	
Study Sites	USA, Australia, Canada, Germany, Italy, Japan, Norway, Spain, Sweden, UK	

2. Study ID Number	NCT04402489<br>**RCT**	PICO
Other Study ID Numbers	MT-7117-G01	
Titel	**Study to Evaluate Efficacy, Safety, and Tolerability of MT-7117 in Subjects With Erythropoietic Protoporphyria or X-Linked Protoporphyria**	
Conditions	Erythropoietic Protoporphyria or X-Linked Protoporphyria	P
Enrolment	184	
Age	12 Years to 75 Years (Child, Adult, Older Adult)	P
Sexes	All	P
Intervention/Treatment	Dersimelagon low dose, Dersimelagon high dose, Placebo	I; C
Phase	3	
Inclusion criteria	1. Subjects provided written informed consent to participate. For minor subjects, both minor assent and parental consent will be provided.<br>2. Male and female subjects with a confirmed diagnosis of EPP or XLP based on medical history, aged 12 years to 75 years, inclusive, at Screening.<br>3. Subjects have a body weight of ≥30 kg.<br>4. Subjects are willing and able to travel to the study sites for all scheduled visits.<br>5. In the Investigator's opinion, subject is able to understand the nature of the study and any risks involved in participation, and willing to cooperate and comply with the protocol restrictions and requirements (including travel).<br>6. Female subjects who are non-lactating and have a negative urine pregnancy test at baseline visit prior to receiving the first dose of study drug.<br>7. Female subjects of childbearing potential and male subjects with partner of child-bearing potential currently using/willing to use 2 effective methods of contraception including barrier method as described in the protocol.	P
Exclusion Criteria:	1. History or presence of photodermatoses other than EPP or XLP.<br>2. Subjects who are unwilling or unable to go outside during daylight hours most days (e.g., between 1 hour post sunrise and 1 hour pre-sunset) during the study.	P/<br><br>Safety

	3. Presence of clinically significant hepatobiliary disease based on LFT values at Screening.<br>4. Subjects with AST, ALT, ALP ≥3.0 × upper limit of normal (ULN) or total bilirubin >1.5 × ULN at Screening.<br>5. Subjects with or having a history (in the last 2 years) of excessive alcohol intake in the opinion of the Investigator.<br>6. History of melanoma.<br>7. Presence of melanoma and/or lesions suspicious for melanoma at Screening.<br>8. History of familial melanoma (defined as having 2 or more first-degree relatives, such as parents, sibling and/or child).<br>9. Presence of squamous cell carcinoma, basal cell carcinoma, or other malignant skin lesions. Any suspicious lesions or nevi will be evaluated. If the suspicious lesion or nevi cannot be resolved through biopsy or excision, the subject will be excluded from the study.<br>10. History or presence of psychiatric disease judged to be clinically significant by the Investigator and which may interfere with the study evaluation and/or safety of the subjects.<br>11. Presence of clinically significant acute or chronic renal disease based upon the subject's medical records including hemodialysis; and a serum creatinine level of greater than 1.2 mg/dL or an estimated glomerular filtration rate (eGFR) <60 ml/min.<br>12. Presence of any clinically significant disease or laboratory abnormality which, in the opinion of the Investigator, can interfere with the study objectives and/or safety of the subjects.<br>13. Female subjects who are pregnant, lactating, or intending to become pregnant during the study.<br>14. Treatment with phototherapy within 3 months before Randomization (Visit 2).<br>15. Treatment with afamelanotide within 3 months before Randomization (Visit 2).<br>16. Treatment with cimetidine within 4 weeks before Randomization (Visit 2).<br>17. Treatment with antioxidant agents within 4 weeks before Randomization (Visit 2), at doses which, in the opinion of the Investigator, may affect study endpoints (including but not limited to beta-carotene, cysteine, pyridoxine).<br>18. Chronic treatment with any scheduled analgesic agents including, but not limited to, opioids and opioid derivatives such as morphine, hydrocodone, oxycodone, fentanyl, or their combination with other unscheduled analgesics or non-steroidal anti-inflammatory drug (Percocet and Vicodin-like prescription drugs) within 4 weeks before Randomization (Visit 2).Acute use of scheduled	

	narcotics greater than 3 months prior to randomization, OTCs, such as NSAIDs or aspirin for analgesia, or prior temporary use of scheduled agents within 3 months of screening are not excluded.<br>19. Treatment with any drugs or supplements which, in the opinion of the Investigator, can interfere with the objectives of the study or safety of the subjects.<br>20. Previous exposure to MT-7117 (this does not include placebo treated subjects).<br>21. Previous treatment with any investigational agent within 12 weeks before Screening OR 5 half-lives of the investigational product (whichever is longer).	
Primary Purpose	Treatment	
Allocation	Randomized	
Interventional Model	Parallel Assignment	
Arms and Interventions	Dersimelagon low dose, Dersimelagon high dose, Placebo	I; C
Masking	Triple (ParticipantCare ProviderInvestigator)	
Primary Outcome Measures	Change from baseline in average daily sunlight exposure time (minutes) to first prodromal symptom (burning, tingling, itching, or stinging) associated with sunlight exposure between 1 hour post sunrise and 1 hour pre-sunset at Week 26. Baseline (Week 0) and 26 weeks	O
Secondary Outcome Measures	Patient Global Impression of Change (PGIC). PGIC: Scale from 1 to 7, where 7 is worse. Week 26	O
	Total number of sunlight-induced pain events with pain rating of 1-10 on the Likert scale during the 26-week double-blind treatment period. Baseline (Week 0) and Week 26	O
Other Outcome Measures	Change from baseline for total score in the domain of pain intensity in the PROMIS-57. Pain intensity: 0 to 10, where 10 is worst pain imaginable. Baseline (Week 0) and Week 26	O
	The percentage of subjects who are responders based on average daily sunlight exposure time to first prodromal symptom associated with sunlight exposure between 1 hour post sunrise and 1 hour pre-sunset defined by within-subject meaningful change. Week 26	O
	Change from baseline for total score in the domain of physical function in the PROMIS-57. Physical function: 1-5, where 5 is without any difficulty. Baseline (Week 0) and Week 26	O
Study Sites	USA, Australia, Canada, Germany, Italy, Japan. Norway, Spain, Sweden, UK	

1Study ID Number	NCT03520036<br>**RCT**	PICO
Other Study ID Numbers	MT-7117-A01	
Titel	**Study to Evaluate Efficacy, Safety, and Tolerability of MT-7117 in Subjects With Erythropoietic Protoporphyria**	
Conditions	Erythropoietic Protoporphyria	P
Enrolment	102	
Age	18 Years to 75 Years (Adult, Older Adult)	P
Sexes	All	P
Intervention/Treatment	• Drug: MT-7117 low dose<br>• Drug: MT-7117 high dose<br>• Drug: Placebo	I; C
Phase	2	
Inclusion criteria	1. Subjects provided written informed consent to participate.<br>2. Male and female subjects with a confirmed diagnosis of EPP based on medical history, aged 18 years to 75 years, inclusive, at Screening.<br>3. Subjects are willing and able to travel to the study sites for all scheduled visits.<br>4. In the Investigator's opinion, subject is able to understand the nature of the study and any risks involved in participation, and willing to cooperate and comply with the protocol restrictions and requirements (including travel).	P
Exclusion Criteria:	1. History or presence of photodermatoses other than EPP.<br>2. Subjects who are unwilling or unable to go outside during daylight hours (e.g., between 1 hour post sunrise and 1 hour pre-sunset) during the study.<br>3. Presence of clinically significant hepatobiliary disease based on LFT values at Screening.<br>4. Subjects with AST, ALT, ALP ≥3.0 × upper limit of normal (ULN) or total bilirubin >1.5 × ULN at Screening.<br>5. Subjects with or having a history (in the last 2 years) of excessive alcohol intake in the opinion of the Investigator.<br>6. History or presence of melanoma and/or atypical nevus at Screening.<br>7. History of familial melanoma (defined as having 2 or more first-degree relatives, such as parents, sibling and/or child).<br>8. History or presence of pre-malignant skin lesion squamous cell carcinoma, basal cell carcinoma, or other malignant skin lesions.	P/ Safety

	9. History or presence of psychiatric disease judged to be clinically significant by the Investigator and which may interfere with the study evaluation and/or safety of the subjects.<br>10. Presence of clinically significant acute or chronic renal disease based upon the subject's medical records including hemodialysis; and a serum creatinine level of greater than 1.2 mg/dL or a glomerular filtration rate (GFR) <60 ml/min.<br>11. Presence of any clinically significant disease or laboratory abnormality which, in the opinion of the Investigator, can interfere with the study objectives and/or safety of the subjects.<br>12. Pregnancy or lactation.<br>13. Females of child bearing potential and male subjects with partners of child-bearing potential unwilling to use adequate contraception measures as described in the protocol.<br>14. Treatment with phototherapy within 3 months before Randomization (Visit 2).<br>15. Treatment with afamelanotide within 3 months before Randomization (Visit 2).<br>16. Treatment with cimetidine within 4 weeks before Randomization (Visit 2).<br>17. Treatment with antioxidant agents at doses which, in the opinion of the Investigator, may affect study endpoints (including but not limited to beta-carotene, cysteine, pyridoxine) within 4 weeks before Randomization (Visit 2).<br>18. Chronic treatment with prescription-based analgesic agents including but not limited to opioids and opioid derivatives such as morphine, hydrocodone, oxycodone or their combination with other analgesics or non-steroidal anti-inflammatory drug (NSAID, as Percocet and Vicodin-like prescription drugs) within 4 weeks before Randomization (Visit 2).<br>19. Treatment with any drugs or supplements which, in the opinion of the Investigator, can interfere with the objectives of the study or safety of the subjects.<br>20. Previous exposure to MT 7117.<br>21. Previous treatment with any investigational agent within 12 weeks before Screening OR 5 half-lives of the investigational product (whichever is longer).	
Primary Purpose	Treatment	
Allocation	Randomized	
Interventional Model	Parallel Assignment	
Arms and Interventions	MT-7117 low dose MT-7117 high dose Placebo	I; C
Masking	Quadruple (ParticipantCare ProviderInvestigatorOutcomes Assessor)	

Primary Outcome Measures	Change From Baseline in Average Daily Time (Minutes) to First Prodromal Symptom Associated With Sunlight Exposure Between Hour Post Sunrise and 1 Hour Pre-Sunset at Week 16.<br><br>Duration in minutes, of sunlight exposure between 1 hour post sunrise and 1 hour pre-sunset. The average Duration in minutes, of sunlight exposure before the first prodromal symptom between 1 hour post sunrise and 1 hour pre-sunset. The average duration means that average of daily durations in 14-day windows before Day 1 (or week 16 Day) applied.	O
	Change From Baseline in Average Daily Duration (Minutes) of Sunlight Exposure Between 1 Hour Post Sunrise and 1 Hour Pre-Sunset Without Prodromal Symptoms at Week 16<br><br>Change from baseline to week 16 in Average Daily Duration (Minutes) of Sunlight Exposure sums any sunlight exposure time excluding any overlapped time with prodromal symptoms, including if the patients go out multiple times on the same day after the prodromal symptom had previously ended.	O
	Change From Baseline in Average Daily Mean Duration (Minutes) of Sunlight Exposure Between 1 Hour Post Sunrise and 1 Hour Pre-Sunset Without Prodromal Symptoms at Week 16<br><br>Change from baseline to week 16 in Average Daily Mean Duration (Minutes) of Sunlight Exposure without prodromal symptoms divided by the number of sunlight exposures periods applicable that day.	O
Secondary Outcome Measures	Total Number of Sunlight Exposure Episodes With Prodromal Symptoms During 16-Week Double-Blind Treatment Period	O
	Change From Baseline in Average Daily Mean Intensity of Prodromal Symptoms During 16-week Double-blind Treatment Period in 11-point Likert Scale<br><br>The Intensity of Prodromal Symptoms is measured by 11-point Likert scale ranges from 0 (no symptom) to 10 (greatest severity of symptom).	O
	Change From Baseline in Average Daily Duration (Minutes) of Prodromal Symptoms at 16-Week Double-Blind Treatment Period	O
	Change From Baseline in Pigmentation as Measured by Melanin Density for Average of 6 Skin Segments at Week 8 and Week 16.	O

	Pigmentation will be assessed in melanin density which are numeric scores measured by spectrophotometer on 6 skin segments (forehead, left cheek, right inside upper arm, left medial forearm, right-hand side of abdomen, and left-hand side of buttock).	
	Percent Change From Baseline in Pigmentation as Measured by Melanin Density for Average of 6 Skin Segments at Week 8 and Week 16.	O
	Total Number of Pain Events During 16-Week Double-Blind Treatment Period	O
Other Outcome Measures	Total Number of Sunlight Exposure Episodes	O
	Change in Pigmentation as Measured by Melanin Density	O
	The Quality of Life as Measured by the Patient Reported Outcomes Measurement Information System (PROMIS) 57	O/ PROM/ QoL
Study Sites	USA	

Table S3c: Clinical trial protocols of pharmacotherapies currently tested in erythropoietic protoporphyria

Bitopertin

3. Study ID Number	NCT05883748<br>**OLE**	PICO
Other Study ID Numbers	DISC-1459-501	
Titel	**Open-Label, Long-Term Extension Study to Investigate the Safety, Tolerability, and Efficacy of DISC-1459 (Bitopertin) in Participants With EPP**	
Conditions	Erythropoietic Protoporphyria (EPP)	P
Age	12 Years and older (Child, Adult, Older Adult)	P
Sexes	All	P
Enrolment	200	
Intervention/Treatment	Bitopertin	I
Phase	2/3	
Inclusion Criteria	• Participants with diagnosis of EPP who are participating (or who have participated) in a prior Disc Medicine bitopertin study and who have completed the randomized treatment phase and End-of-Study visit<br>• Aged ≥12 years upon study consent<br>• Body weight ≥32 kg for participants <18 years of age and BMI ≥18.5 kg/m2 for adult participants	P
Exclusion Criteria	• Participants who have an ongoing SAE from a clinical study that is assessed by the investigator as related to bitopertin<br>• Other medical or psychiatric condition or laboratory finding not specifically noted above that, in the judgement of the Investigator or Sponsor, would put the participant at unacceptable risk or otherwise preclude participation in the study<br>• Condition or concomitant medication that would confound the ability to interpret clinical, clinical laboratory, or participant diary data, including a major psychiatric condition that has had an exacerbation or required hospitalization in the last 6 months<br>• Planned treatment with afamelanotide or dersimelagon during the study<br>• Planned use of any drugs or herbal remedies known to be strong inhibitors or inducers of cytochrome p450 (CYP)3A4 enzymes throughout the study<br>• If female, pregnant, or breastfeeding	P/<br><br>Safety

	• Participation in any other clinical protocol or investigational trial, other than Disc Medicine bitopertin trials, that involves administration of experimental therapy and/or therapeutic devices within 30 days of Day 1<br>• Score of PHQ-8 ≥10 at screening or any response of "yes" on the C-SSRS<br>• Grapefruit/Seville orange and products containing these for 14 days prior to first dose of study drug and throughout the study	
Primary Purpose	Treatment	
Allocation	Randomized	
Interventional Model	Parallel Assignment	
Arms and Interventions	Bitopertin dose 1 and dose 2	I
Masking	None (Open Label)	C
Primary Outcome Measures	up to 5 Years: Incidence of treatment-emergent adverse events	
	up to 5 Years: Incidence of clinically abnormal vital signs	
	up to 5 Years: Incidence of clinically abnormal physical exam	
	up to 5 Years: Incidence of abnormal laboratory test results	
	up to 5 Years: Assessment of Patient Health Questionnaire (PHQ-8): The Patient Health Questionnaire (PHQ-8), an 8-item participant-report measure for screening for depression and for establishing depression severity. The total score ranges from 0-24, with a higher score indicating greater depression symptom severity	Safety
	up to 5 Years: Assessment of C-SSRS: The C-SSRS is a clinician-rated scale that assesses suicidality from ideation to behaviors and monitors the potential emergence of suicidality in clinical studies.	Saftey
Secondary Outcome Measures	up to 5 Years:<br>Change from baseline in daily daylight tolerance, as assessed by total hours spent in the sunlight without pain and average time to first prodromal syndrome in sunlight	O
	up to 5 Years: Change from baseline in whole blood metal-free PPIX levels	O
	up to 5 Years: Plasma Bitopertin Concentrations	
Study Sites	USA, Australia	

2. Study ID Number	NCT05308472 – trial used for the analysis --<br>**RCT**	PICO
Other Study ID Numbers	DISC-1459-201, AURORA	
Titel	**Study of Bitopertin to Evaluate the Safety, Tolerability, Efficacy, and PPIX Concentrations in Participants With EPP**	
Conditions	Erythropoietic Protoporphyria (EPP)	P
Age	18 Years and older (Adult, Older Adult)	P
Sexes	All	P
Enrolment	75	
Intervention/Treatment	Bitopertin	I
Phase	2	
Inclusion Criteria	1. Aged 18 years or older at the time of signing the informed consent form (ICF).<br>2. Diagnosis of EPP, based on medical history by ferrochelatase (FECH) genotyping or by biochemical porphyrin analysis.<br>3. Body weight ≥50 kg.<br>4. Washout of at least 2 months prior to Screening of afamelanotide and dersimelagon, if applicable.<br>5. Aspartate aminotransferase (AST) and alanine transaminase (ALT) <2× upper limit of normal (ULN) and total bilirubin <ULN (unless documented Gilbert syndrome) at Screening. Albumin >lower limit of normal (LLN).	P/<br><br>Safety
Exclusion Criteria	1. Major surgery within 8 weeks before Screening or incomplete recovery from any previous surgery.<br>2. Other than EPP, an inherited or acquired red cell disease associated with anemia.<br>3. A history or known allergic reaction to any investigational product excipients or history of anaphylaxis to any food or drug.<br>4. History of liver transplantation.<br>5. History of alcohol dependence or excessive alcohol consumption, as assessed by the Investigator.<br>6. Human immunodeficiency virus (HIV), active Hepatitis B, or C.<br>7. Other medical or psychiatric condition or laboratory finding not specifically noted above that, in the judgment of the Investigator or Sponsor, would put the participant at unacceptable risk or otherwise preclude the participant from participating in the study<br>8. Condition or concomitant medication that would confound the ability to interpret clinical, clinical laboratory, or participant diary data, including a major psychiatric	P/<br><br>Safety

	condition that has had an exacerbation or required hospitalization in the last 6 months.<br><br>Treatment History:<br><br>9. Concurrent or planned treatment with afamelanotide or dersimelagon during the study period.<br>10. Treatment with opioids for any period >7 days in the 2 months prior to screening or anticipated to require opioid use for >7 days at any point during the study.<br>11. New treatment for anemia, including initiation of iron supplementation, in the 2 months prior to Screening.<br>12. Current or planned use of any drugs or herbal remedies known to be strong inhibitors or inducers of CYP3A4 enzymes for 28 days prior to the first dose and throughout the study.<br><br>Laboratory Exclusions:<br><br>13. Hemoglobin <10 g/dL at Screening.	
Primary Purpose	Treatment	
Allocation	Randomized	
Interventional Model	Parallel Assignment	
Arms and Interventions	Placebo: Oral dose, once a day for 120 days<br>Bitopertin: Oral dose level 1, once a day for 120 days<br>Bitopertin: Oral dose level 2, once a day for 120 days<br>Bitopertin: Oral dose level 1, once a day for up to 8 months	C; I
Masking	Triple (Participant Investigator Outcomes Assessor)	
Primary Outcome Measures	Percent change from baseline in whole blood metal-free PPIX levels: 121 days	O
Secondary Outcome Measures	121 days:<br>Total hours of sunlight exposure to skin on days with no pain from 1000 to 1800 hours (10:00am to 6:00pm)	O
	Daily sunlight exposure time (minutes) to first prodromal symptom (burning, tingling, itching, or stinging) associated with sunlight exposure between 1 hour post-sunrise and 1 hour pre-sunset	O
	Pain intensity of phototoxic reactions according to a Likert scale (0-10)	O
	Incidence of treatment-emergent adverse events	O

	Erythrocyte total PPIX concentrations	O
	Plasma total PPIX concentrations	O
	Whole blood total PPIX concentrations	O
	Plasma bitopertin concentrations	
Other Outcome Measures	Plasma maximum measured drug concentration (Cmax)	
	Time of maximum concentration (Tmax)	
	Area under the concentration-time curve (AUC)	
Study Sites	USA	

1. Study ID Number	ACTRN12622000799752<br>**Open label study**	PICO
Source	anzctr.org.au	
Other Study ID Numbers	BEACON; DISC-1459-202	
Titel	**A Phase 2, Randomized, Open Label Study of Bitopertin to Evaluate the Safety, Tolerability, Efficacy, and Protoporphyrin IX (PPIX) Concentrations in Participants with Erythropoietic Protoporphyria (EPP)**	
Conditions	Erythropoietic Protoporphyria (EPP)	P
Age	18 and older	P
Sexes	Both males and females	P
Enrolment	22	
Intervention/Treatment	Bitopertin	I
Phase	II	
Inclusion Criteria	1. Aged 18 years or older<br><br>2. Diagnosis of EPP or XLP, based on medical history of FECH or ALAS2 genotyping or by biochemical porphyrin analysis.<br><br>3. Body weight greater than or equal to 50 kg.<br><br>4. Washout of at least 2 months prior to Screening of afamelanotide or dersimelagon, if applicable.	P/<br><br>Safety

	5. Aspartate aminotransferase (AST) and alanine transaminase (ALT) less than 2×upper limit of normal (ULN) and total bilirubin less than ULN (unless documented Gilbert syndrome) at Screening. Albumin greater than lower limit of normal (LLN).<br><br>6. If male with female sexual partner(s) of childbearing potential, agrees he and partner will use one of the following acceptable methods of birth control during the study and for 30 days after the last study drug dose:<br><br>a. abstinence<br><br>b. stable hormonal contraceptive or a barrier method (e.g., condom [male or female] or diaphragm)<br><br>c. intrauterine device, in place for at least 3 months<br><br>d. surgically sterile by hysterectomy, bilateral oophorectomy, or bilateral tubal ligation<br><br>7. If female of childbearing potential, defined as prior menarche, no hysterectomy, no bilateral oophorectomy, not postmenopausal (at least 12 months natural, spontaneous amenorrhea), must commit to one of the following methods of acceptable birth control during the study and for 30 days after the last study drug dose:<br><br>a. abstinence<br><br>b. stable hormonal contraceptive in conjunction with a barrier method (e.g., condom [male or female] or diaphragm)<br><br>c. intrauterine device, in place for at least 3 months<br><br>8. Negative urine or serum pregnancy test (females of childbearing potential) at Screening (Days -28 to -1) AND Baseline (Day 1), prior to dosing.<br><br>9. Able to understand the study aims, procedures, and requirements, and provide written informed consent.	

	10. Able to comply with all study procedures.	
Exclusion Criteria	1. Major surgery within 8 weeks before Screening or incomplete recovery from any previous surgery.<br><br>2. Other than EPP, an inherited or acquired red cell disease associated with anemia.<br><br>3. A history or known allergic reaction to any investigational product excipients or history of anaphylaxis to any food or drug.<br><br>4. History of liver transplantation.<br><br>5. History of alcohol dependence or excessive alcohol consumption, as assessed by the Investigator.<br><br>6. Human immunodeficiency virus (HIV), active Hepatitis B, or C. A positive Hepatitis B result, indicating active disease status, should be discussed between the Investigator and Sponsor prior to enrollment.<br><br>7. Other medical or psychiatric condition or laboratory finding not specifically noted above that, in the judgment of the Investigator or Sponsor, would put the participant at unacceptable risk or otherwise preclude the participant from participating in the study<br><br>8. Condition or concomitant medication that would confound the ability to interpret clinical, clinical laboratory, or participant diary data, including a major psychiatric condition that has had an exacerbation or required hospitalization in the last 6 months.<br><br>9. Concurrent or planned treatment with afamelanotide or dersimelagon.<br><br>10. Treatment with opioids for any period greater than 7 days in the 2 months prior to screening or anticipated to require opioid use for greater than 7 days at any point during the study.<br><br>11. Treatment for anemia, including iron supplementation, in the 2 months prior to Screening.	P/<br><br>Safety

	12. Current or planned use of any drugs or herbal remedies known to be strong inhibitors or inducers of CYP3A4 enzymes for 28 days prior to the first dose and throughout the study<br><br>13. Hemoglobin less than 10 g/dL at Screening.<br><br>14. If female, pregnant or breastfeeding.<br><br>15. Participation in any other clinical protocol or investigational trial that involves administration of experimental therapy and/or therapeutic devices within 30 days of Screening.<br><br>16. Grapefruit/Seville orange and food products containing these, for 14 days prior to first dose and throughout the study	
Primary Purpose	The purpose of this research is to assess the safety, tolerability and effectiveness of bitopertin in patients with EPP and XLP. This will be the first time it has been given to individuals with EPP/XLP. In this study, up to 22 patients will be enrolled in Australia only. Erythropoietic protoporphyria and XLP are rare diseases which cause severe light sensitivity. Both are caused by mutations in the genes that produce enzymes of the heme production pathway. These defects lead to abnormally high levels of protoporphyrin IX (PPIX) in red blood cells, which leads to painful reactions when exposed to light. Additionally, PPIX accumulates in the liver and gall bladder and can cause gallstones and liver impairment.<br>Current therapies for EPP/XLP are aimed at managing symptoms. At present, none act to reduce the high levels of PPIX in the blood. Increased PPIX is thought to be the underlying cause of the severe and potentially life-threatening effects of EPP/XLP. Thus, there remains a significant unmet medical need for new therapies which address the underlying causes of the disease.<br>Disc Medicine Inc is developing bitopertin, an inhibitor of glycine transporter-1 (GlyT1) as a new treatment for EPP/XLP.	O
Allocation	n.a.	
Interventional Model	n.a.	
Arms and Interventions	n.a.	
Masking	Open label	C
Primary Outcome Measures	n.a.	
Secondary Outcome Measures	n.a.	

Other Outcome Measures	n.a.	
Study Sites	Australia	

Table S3c: Clinical trial protocols of pharmacotherapies currently tested in erythropoietic protoporphyria

Cimetidine

1. Study ID Number	NCT05020184 – trial used for the analysis --	PICO
Other Study ID Numbers	2021P002095	
Titel	Effect of Oral Cimetidine in the Protoporphyrias	
Conditions	Erythropoietic protoporphyria (EPP) and X-linked protoporphyria (XLP)	P
Age	15 Years and older (Child, Adult, Older Adult)	P
Sexes	All	P
Enrolment	20	
Intervention/Treatment	Oral Cimetidine	I
Phase	2	
Inclusion Criteria	• Prior enrollment or co-enrollment in the Longitudinal Study of the Porphyrias (PC Study 7201) with a confirmed diagnosis of EPP or XLP<br>• Male or female age ≥15 years at screening<br>• Characteristic history of non-blistering cutaneous photosensitivity<br>• Willing and capable of giving informed consent and following procedures described in the protocol	P
Exclusion Criteria	• Participants not willing to expose themselves to light to the point of prodromal symptoms at least weekly<br>• History of liver or bone marrow transplant or clinically significant liver dysfunction as determined by the Investigator<br>• Known or suspected allergy or intolerance to cimetidine<br>• Use of any other experimental therapy in the past 3 months at screening<br>• Use of cimetidine within the past 3 months at screening<br>• Individuals with elevations of porphyrins in plasma or erythrocytes due to other diseases (i.e., secondary porphyrinemia) such as liver and bone marrow diseases<br>• Patients with any clinically significant comorbid conditions, which in the opinion of the Investigator, precludes participation<br>• Treatment with any drugs or supplements (Appendix 1) that in the opinion of the Investigator can interfere with subject safety or the objectives of the study	P/<br><br>Safety

	• The participant either does not have a smartphone or is not willing to use his/her smartphone for the study<br>• Women who are pregnant, breastfeeding, or actively planning to become pregnant<br>• Individuals with moderate to severe renal insufficiency	
Primary Purpose	Treatment	
Allocation	Randomized	
Interventional Model	Crossover Assignment.	
Arms and Interventions	The study design is a multicenter, prospective, randomized, double-blind, placebo-controlled, crossover trial of oral cimetidine 800mg twice daily versus placebo	I
Masking	Quadruple (Participant Care Provider Investigator Outcomes Assessor) randomized, double-blind, placebo-controlled	
Primary Outcome Measures	Erythrocyte total protoporphyrin level: Percent change in erythrocyte total protoporphyrin level post-treatment relative to pre-treatment, Before and after each 3-month treatment period	O
Secondary Outcome Measures	Time to prodrome: Time to prodrome measured as prodrome-free outdoor exposure time, Last 2 months of each treatment period	O
	Patient-reported quality of life: Patient-reported quality of life as measured by Patient-Reported Outcomes Measurement Information System-57 (PROMIS-57) scale [0-100, where 100 is the best quality of life] Before and after each 3-month treatment period	O/ PROM/ QoL
	Phototoxic episodes: The number and severity of sunlight-induced pain events (phototoxic episodes). Last 2 months of each treatment period	O
	Light dose: Light dose required for time to prodrome. Last 2 months of each treatment period	O
Study Sites	USA	
