# Supplementary material for: New pharmacotherapies for the erythropoietic protoporphyrias: an analysis of trial protocols from a patient perspective
Source: Orphanet J Rare Dis. 2025 Dec 29;20:637. doi: 10.1186/s13023-025-04170-9 (PMC12751534; doi:10.1186/s13023-025-04170-9)
Supplement: Supplementary file 2 — Supplementary Material 2 [file 13023_2025_4170_MOESM2_ESM.pdf]

Supplement 2, Table S4

New pharmacotherapies for the erythropoietic protoporphyrias. An analysis of the trial protocols from a patient perspective

Dechant et al.

Table S4: Comparison of the trial protocols of the four currently investigated pharmacotherapies for treating EPP p.2-8

Supplement 2: New pharmacotherapies for the erythropoietic protoporphyrias. An analysis of the trial protocols from a patient perspective; Dechant et al.

Table S4: Comparison of the trial protocols of the four currently investigated pharmacotherapies for treating EPP

Table S4 is the full version of the result summary table 1 in the manuscript

	**Afamelanotide**	**Dersimelagon**	**Bitopertin**	**Cimetidine**
**Clinical trials**<br>- phase<br>- n, duration<br>- additional information				
Trial with the most comprehensive information available	CUV039:<br>- phase III, pivotal study<br>- n=93, 180 days<br>- RCT<br>- full protocol and results published [26]	MT-7117-A-302:<br>- phase III<br>- n=150, 16 weeks<br>- RCT	DISC-1459-201:<br>- phase II<br>- n=75, 121 days<br>- RCT	2021P002095:<br>- phase II<br>- n=20, 3 months<br>- RCT, crossover
Other clinical trials	CUV-PASS-001:<br>- phase IV<br>- n=200-660, ongoing<br>- OL<br>- full protocol and results for the Dutch cohort published [29]<br><br>CUV052:<br>- phase I/II pharmacokinetics<br>- n=28, 90 days<br>- OL	MT-7117-A01:<br>- phase II<br>- n=102, 16 weeks<br>- RCT<br>- full protocol and results published [52]<br><br>MT-7117-G01:<br>- phase III<br>- n=184, 26 weeks<br>- RCT<br><br>MT-7117-A-301:<br>- OLE<br>- n=151, ongoing	DISC-1459-202:<br>- phase II<br>- n=22, [&] 24 weeks [54]<br>- OL<br><br>DISC-1459-501:<br>- OLE<br>- ongoing	n.a.
**3.3.1. Population (inclusion and exclusion criteria)**				
3.3.1.1. Subtypes (EPP1, EPP2, XLEPP)	Male or female subjects with characteristic symptoms of EPP	Male and female subjects with a confirmed diagnosis of EPP or XLP	Diagnosis of EPP, based on medical history by ferrochelatase (FECH)	Prior enrollment or co-enrollment in the Longitudinal Study of the

	phototoxicity and a biochemically-confirmed diagnosis of EPP.	based on medical history.	genotyping or by biochemical porphyrin analysis	Porphyrias (PC Study 7201) with a confirmed diagnosis of EPP or XLP
3.3.1.2.<br>Age range	≥18<br><br>CUV052 (PK):<br>≥12 to 70	≥12 to 75	≥18<br><br>DISC-1459-201:<br>≥ 18<br>[&] ≥12 [54]<br><br>DISC-1459-501 (OLE):<br>≥12	≥15
3.3.1.3.<br>Sex	Male and female	Male and female	Male and female	Male or female
3.3.1.4.<br>Requirement to cease treatment with afamelanotide		Exclusion criterion:<br>Treatment with any of the following medications or therapy within each period before Randomization (Visit 2); Afamelanotide within 3 months	Exclusion criterion:<br>Concurrent or planned treatment with afamelanotide or dersimelagon during the study period.<br><br>Inclusion criterion:<br>Washout of at least 2 months prior to Screening of afamelanotide and dersimelagon, if applicable.	Exclusion criterion:<br>Treatment with any drugs or supplements (Appendix 1) that in the opinion of the Investigator can interfere with subject safety or the objectives of the study.<br><br>[Appendix 1 is not provided]
3.3.1.5.<br>Requirement to expose to sunlight during the clinical trial		Subjects who are unwilling or unable to go outside in sunlight during daylight hours most days (e.g., between 1-hour post-sunrise and 1 hour pre-sunset) during the study.		Exclusion criterion:<br>Participants not willing to expose themselves to light to the point of prodromal* symptoms at least weekly
3.3.1.6.<br>Liver health	Exclusion criterion:	Exclusion criterion:	Inclusion criterion:<br>Aspartate	Exclusion criterion:<br>History of liver or bone

	EPP patients with significant hepatic involvement	Subjects with aspartate aminotransferase (AST), alanine aminotransferase (ALT), alkaline phosphatase (ALP) ≥ 2.0 × upper limit of normal (ULN) or total bilirubin >1.5 × ULN at Screening.	aminotransferase (AST) and alanine transaminase (ALT) <2× upper limit of normal (ULN) and total bilirubin <ULN (unless documented Gilbert syndrome) at Screening. Albumin >lower limit of normal (LLN).	marrow transplant or clinically significant liver dysfunction as determined by the Investigator
3.3.1.7.<br>Skin cancers	Exclusion criterion:<br>Personal history of melanoma or dysplastic nevus syndrome.<br><br>Exclusion criterion:<br>Current Bowen's disease, basal cell carcinoma, squamous cell carcinoma, or other malignant or premalignant skin lesions.	Exclusion criterion:<br>History of melanoma.<br><br>Exclusion criterion:<br>Presence of squamous cell carcinoma, basal cell carcinoma, or other malignant skin lesions. Any suspicious lesions or nevi will be evaluated. If the suspicious lesion or nevi cannot be resolved through biopsy or excision, the subject will be excluded from the study.		
3.3.1.8.<br>Anemia			Exclusion criterion:<br>Hemoglobin <10 g/dL at Screening	
3.3.1.9.<br>Depression and suicidal ideations			Exclusion criterion:<br>DISC-1459-501 (OLE): Score of PHQ-8 ≥10 at screening or any response of "yes" on the C-SSRS	
3.2.1.10.		Exclusion criterion:<br>Subjects who participated in any		Exclusion criterion:

Previous exposure to study drug		previous MT-7117 clinical studies		Use of cimetidine within the past 3 months at screening
**3.3.2. Intervention**				
3.3.2.1. Substance class	Peptide	Small molecule	Small molecule	Small molecule
3.3.2.2. Effect	Symptomatic	Symptomatic	Causative	Causative
3.3.2.3. Mode of action	& Binding to the melanocortin 1 receptor (MC1R): Increase in eumelanin (brown skin pigment) synthesis and strong anti-inflammatory properties [53]	& Binding to MC1R: Increase in eumelanin synthesis [52]<br>*In-vitro* results suggest anti-inflammatory properties [53]	& Inhibitor of glycine transporter 1: Decrease of intracellular concentration of glycine, a PPIX substrate [45]	& Assumed mode of action: Inhibition of ALAS2, the rate-limiting enzyme of the erythroid heme biosynthesis [55]<br>*In-vitro* results do not support mode of action [72]
3.3.2.4 Administration	& 16 mg slow-release subcutaneous implant formulation, every 60 days, approved formulation [26]	Oral, once daily	Oral, once a day<br><br>MT-7117-A-301:<br>200 mg	Oral, twice daily<br><br>2021P002095:<br>800 mg, twice daily
**3.3.3. Comparator**				
3.3.3.1. Comparator main trial	Placebo	Placebo	Placebo	Placebo, crossover study
**3.3.4. Outcomes**				
3.3.4.1. Primary outcome measure(s)	Duration of Direct Sunlight Exposure Between 10:00 and 18:00 Hours on Days When no Pain Was	Change from baseline in average daily sunlight exposure time (minutes) to first prodromal* symptom (burning, tingling, itching, or	Percent change from baseline in whole blood metal-free PPIX levels: 121 days<br><br>DISC-1459-501 (OLE):	Erythrocyte total protoporphyrin level: Percent change in erythrocyte total protoporphyrin level post-treatment relative to

	Experienced (Pain Score of 0).<br>The amount of direct sunlight exposure between 10:00 and 18:00 hours on days when no pain was experienced (e.g.11-point Likert pain score of 0). Time was recorded in a patient diary using 15 minute time blocks.<br>The pain score is measured by the 11-point Likert Pain scale with minimum of 0 and maximum of 10.<br>Likert Pain scale of 0 represents no pain and 10 represents worst imaginable pain.<br>Daily for 6 months	stinging) associated with sunlight exposure between 1 hour post-sunrise and 1 hour pre-sunset at Week 16. The comparison between MT-7117 treatment group and placebo group will be performed. Week 16	up to 5 Years: Assessment of Patient Health Questionnaire (PHQ-8): The Patient Health Questionnaire (PHQ-8), an 8-item participant-report measure for screening for depression and for establishing depression severity. The total score ranges from 0-24, with a higher score indicating greater depression symptom severity<br><br>DISC-1459-501 (OLE):<br>up to 5 Years: Assessment of C-SSRS: The C-SSRS is a clinician-rated scale that assesses suicidality from ideation to behaviors and monitors the potential emergence of suicidality in clinical studies.	pre-treatment, Before and after each 3-month treatment period
3.3.4.2. Secondary outcome measure(s)	Combined Sun Exposure and Phototoxic Pain<br><br>Duration of direct sunlight exposure between 10:00 and 18:00 hours during the study.<br><br>Photoprovocation: A subset of subjects was photoprovoked on the	Total number of sunlight-induced pain events defined as prodromal* symptoms (burning, tingling, itching, or stinging) with pain rating of 1-10 on the Likert scale during the 16-week double-blind treatment period. The comparison between MT-7117 treatment group and	121 days: Total hours of sunlight exposure to skin on days with no pain from 1000 to 1800 hours (10:00am to 6:00pm)<br><br>Daily sunlight exposure time (minutes) to first prodromal* symptom (burning, tingling, itching, or stinging) associated with sunlight exposure	Time to prodrome*: Time to prodrome measured as prodrome-free outdoor exposure time, Last 2 months of each treatment period<br><br>Phototoxic episodes: The number and severity of sunlight-induced pain events (phototoxic

	dorsal surface of the hand (predilection place) and lower back and the minimum symptom dose (MSD) determined on Days 0, 30, 60, 90 and 120.The amount of radiation required to provoke the first clinical symptom was recorded.<br><br>Maximum Severity of Phototoxic Reaction Experienced by Participants.<br><br>Total Number Phototoxic Reactions Experienced by Participants. Daily for 6 months	placebo group will be performed. Week 16<br><br>Total number of sunlight-induced non-prodrome*, phototoxic reactions during the 16-week double-blind treatment period. The comparison between MT-7117 treatment group and placebo group will be performed. Week 16	between 1 hour post-sunrise and 1 hour pre-sunset<br><br>Pain intensity of phototoxic reactions according to a Likert scale (0-10)<br><br>Incidence of treatment-emergent adverse events<br><br>Erythrocyte total PPIX concentrations<br><br>Plasma total PPIX concentrations<br><br>Whole blood total PPIX concentrations	episodes). Last 2 months of each treatment period<br><br>Light dose: Light dose required for time to prodrome*. Last 2 months of each treatment period
3.3.4.3.<br>QoL and PROM measures	The Quality of life of participant is measured using DLQI and EPP QoL.	Patient Global Impression of Change (PGIC) at Week 16. The comparison between MT-7117 treatment group and placebo group will be performed. Week 16<br><br>MT-7117-A01:<br>The Quality of Life as Measured by the Patient Reported Outcomes Measurement Information System (PROMIS) 57	[&] Patient Global Impression of Change [52]	Patient-reported quality of life: Patient-reported quality of life as measured by Patient-Reported Outcomes Measurement Information System-57 (PROMIS-57) scale [0-100, where 100 is the best quality of life] Before and after each 3-month treatment period

* Prodromal symptoms are early warning signals for a phototoxic reaction, and include symptoms such as itching, tingling, burning and stinging; EPP-QoL: EPP Quality of Live; DLQI: Dermatology Life Quality Index; SF-36: Short Form 36 Health Survey; PGIC: Patient Global Impression of Change; PROMIS-57: Patient-Reported Outcomes Measurement Information System; [&] Information supplemented from other sources than clinical trial registries.
